# Supplementary material for: Chemical and Biological Evaluation of Novel 1H-Chromeno[3,2-c]pyridine Derivatives as MAO Inhibitors Endowed with Potential Anticancer Activity
Source: Int J Mol Sci. 2023 Apr 23;24(9):7724. doi: 10.3390/ijms24097724 (PMC10178506; doi:10.3390/ijms24097724)
Supplement: Supplementary file 1 [file ijms-24-07724-s001.zip › ijms-2339348-supplementary.pdf]

Supplementary Materials

Chemical and Biological Evaluation of Novel 1*H*-Chromeno[3,2-*c*]pyridine Derivatives as MAO Inhibitors Endowed with Potential Anticancer Activity

Larisa N. Kulikova [1,†], Rosa Purgatorio [2,†], Andrey A. Beloglazkin [3], Viktor A. Tafeenko [4], Raesi Gh. Reza [1], Daria D. Levickaya [1], Sabina Sblano [2], Angelina Boccarelli [5], Modesto de Candia [2], Marco Catto [2], Leonid G. Voskressensky [1] and Cosimo D. Altomare [2,*]

[1] Organic Chemistry Department, Peoples' Friendship University of Russia (RUDN University), 6 Miklukho-Maklaya St., 117198 Moscow, Russia; raesighulamreza@gmail.com (R.G.R.)

[2] Department of Pharmacy-Pharmaceutical Sciences, University of Bari Aldo Moro, Via E. Orabona 4, 70125 Bari, Italy

[3] A.V. Topchiev Institute of Petrochemical Synthesis, Russian Academy of Sciences, 29 Leninskiy Prosp., 119991 Moscow, Russia; aabeloglazkin@mail.ru

[4] Department of Chemistry, Lomonosov Moscow State University, Leninskie Gory 1-3, 119234 Moscow, Russia; tafeenko-victor@yandex.ru

[5] Department of Precision and Regenerative Medicine and Ionian Area, School of Medicine, University of Bari Aldo Moro, Piazza Giulio Cesare 11, 70124 Bari, Italy

* Correspondence: cosimodamiano.altomare@uniba.it; Tel.: +39-080-5442781

† These authors contributed equally to this work

Table of Content

1. ^{1}H and ^{13}C NMR spectra of compounds **2–8**

2. Single crystal X-ray analyses of compound **3a**, **7c** and **8a**

Figure S1. *¹H NMR data of* ***2a***

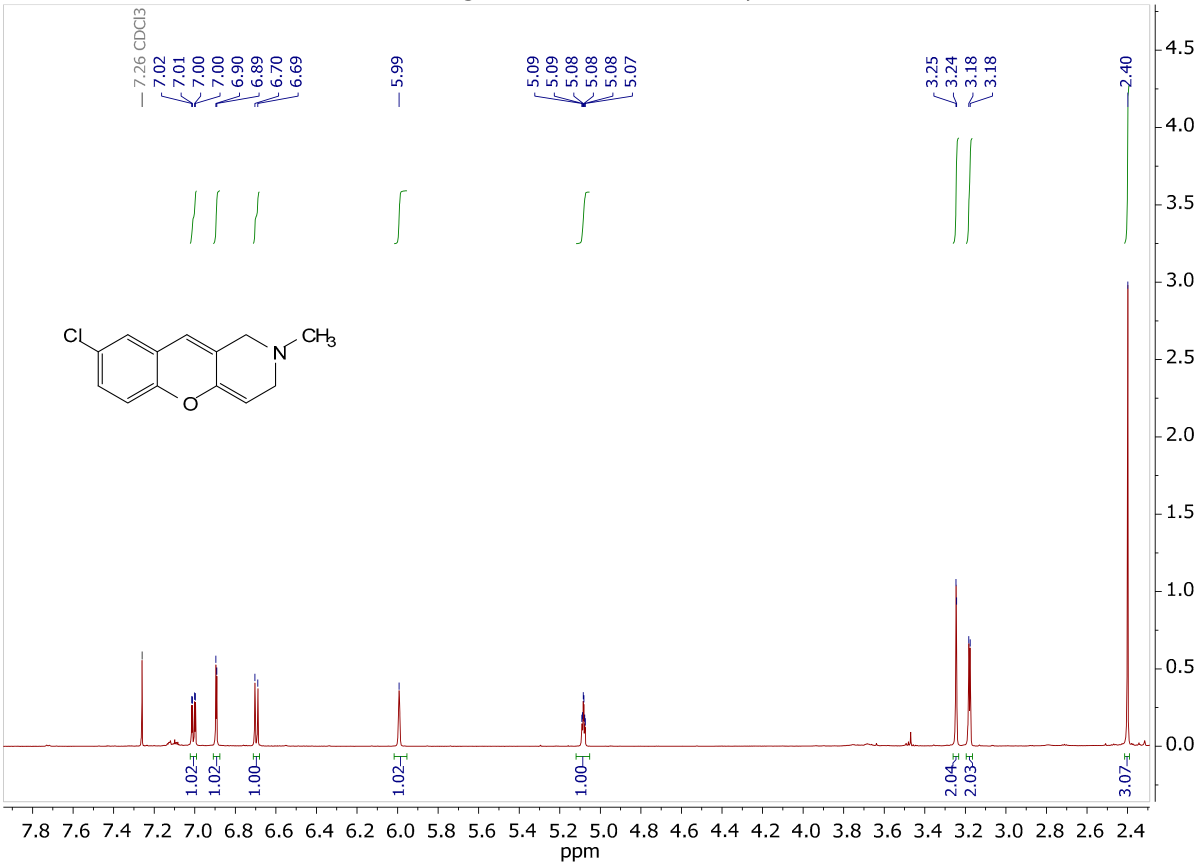

Figure S2. *^{13}C NMR data of* ***2a***

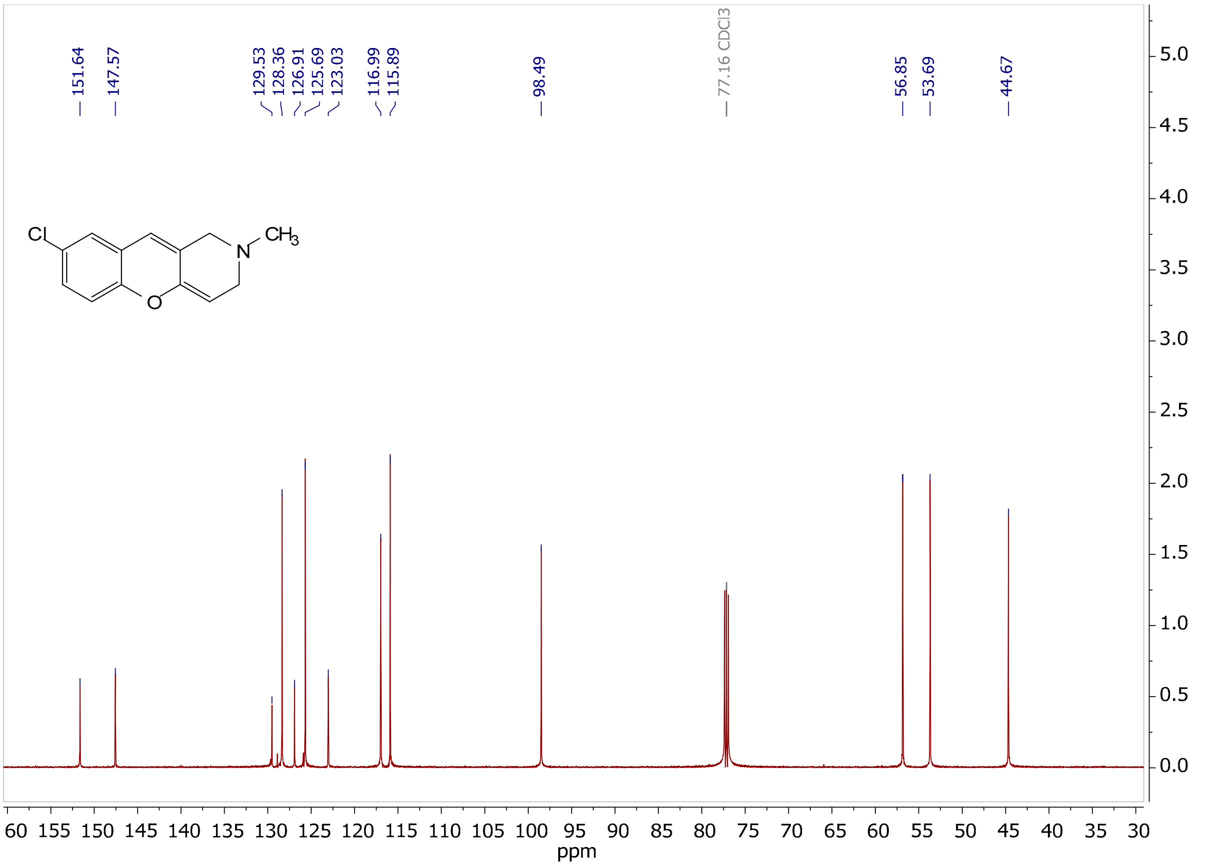

Figure S3. *¹H NMR data of* ***2b***

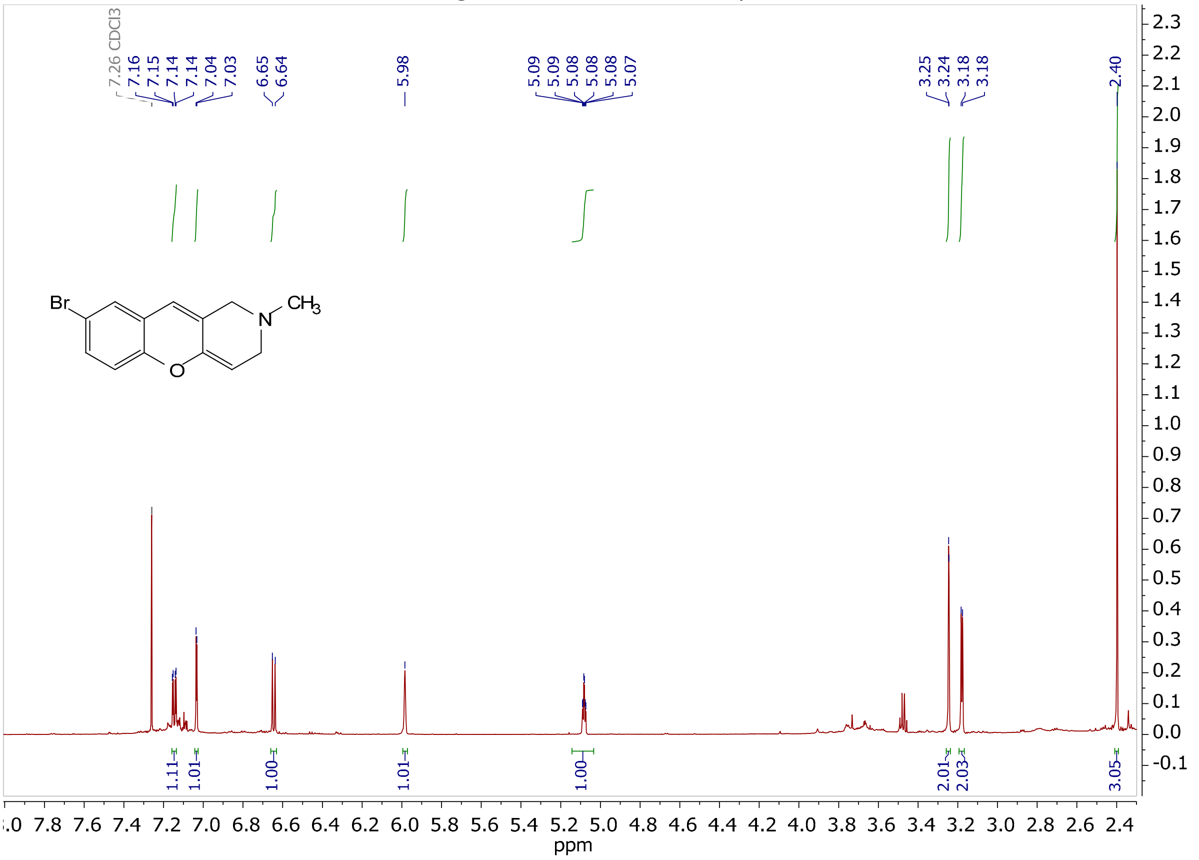

Figure S4. *¹³C NMR data of* ***2b***

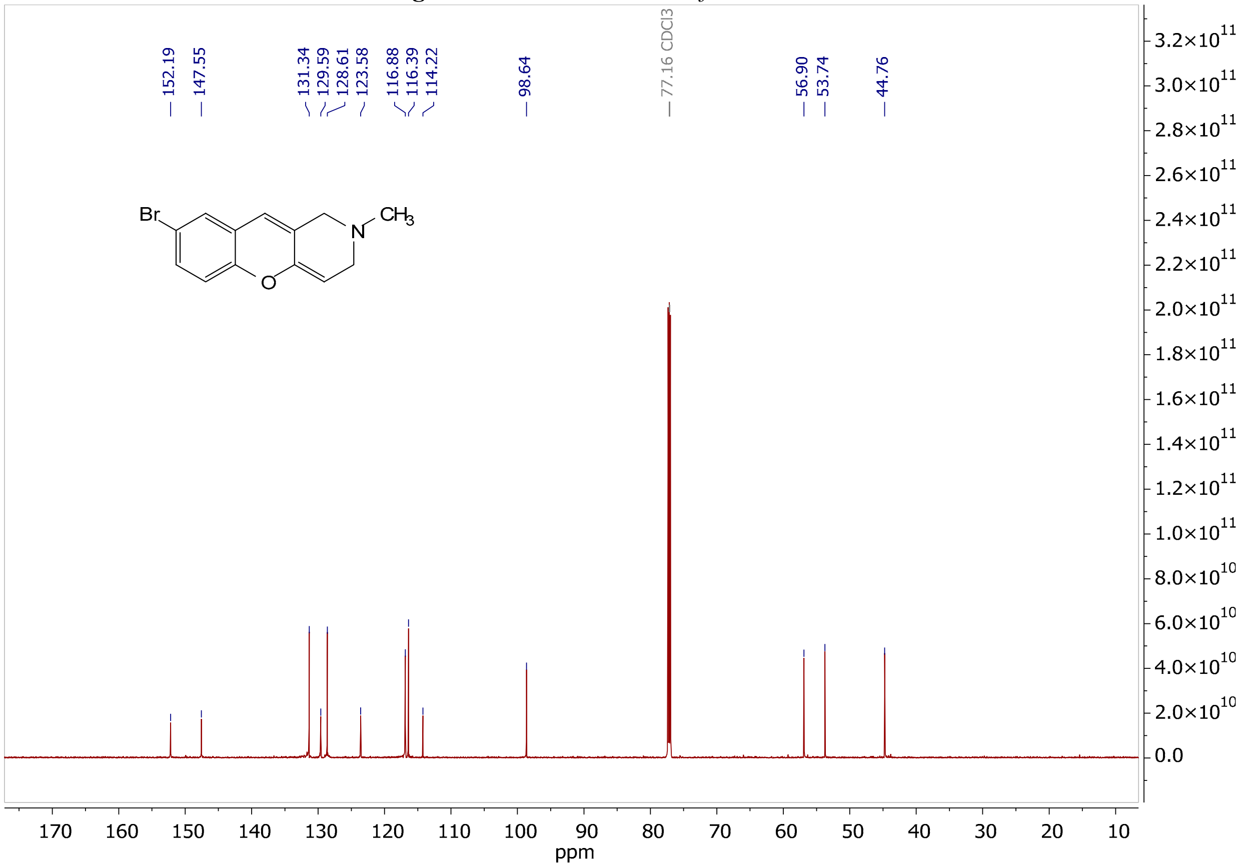

Figure S5. ^{1}H NMR data of ***3a***

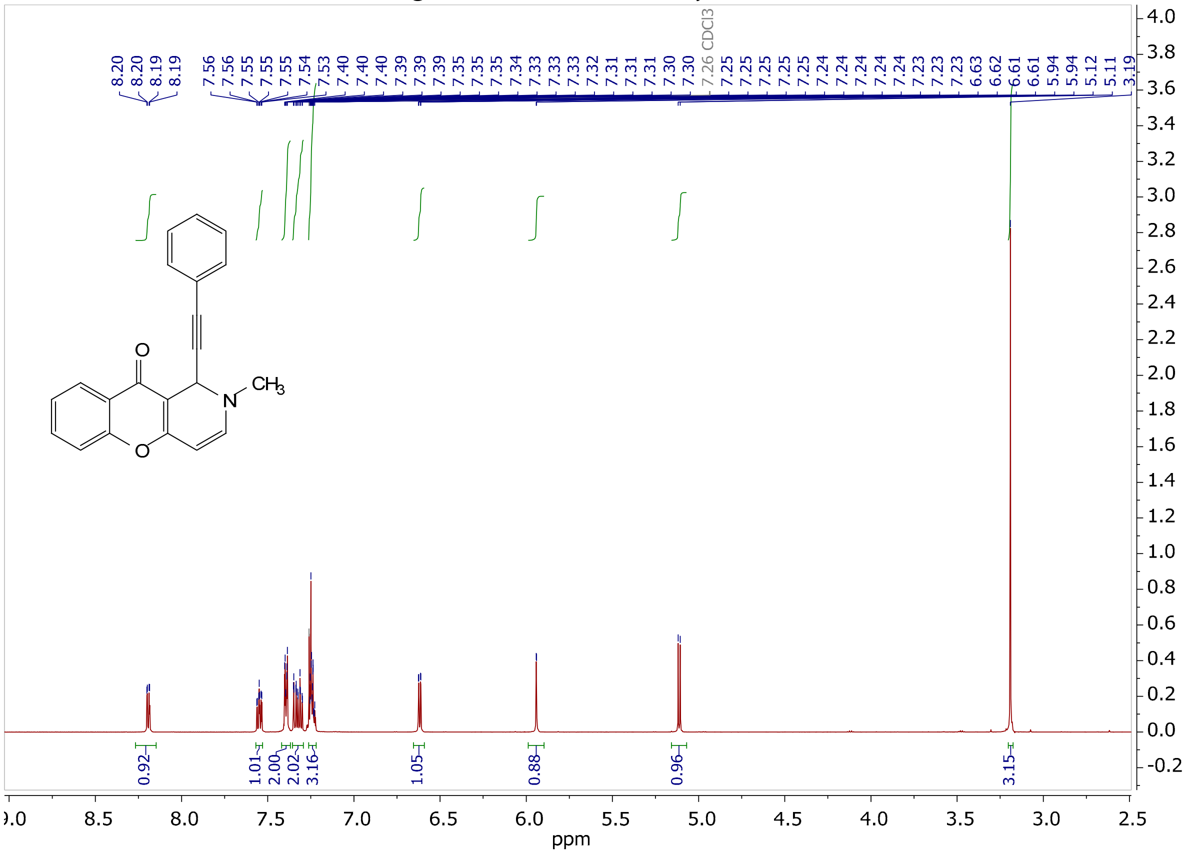

Figure S6. ^{1}H NMR data of ***3b***

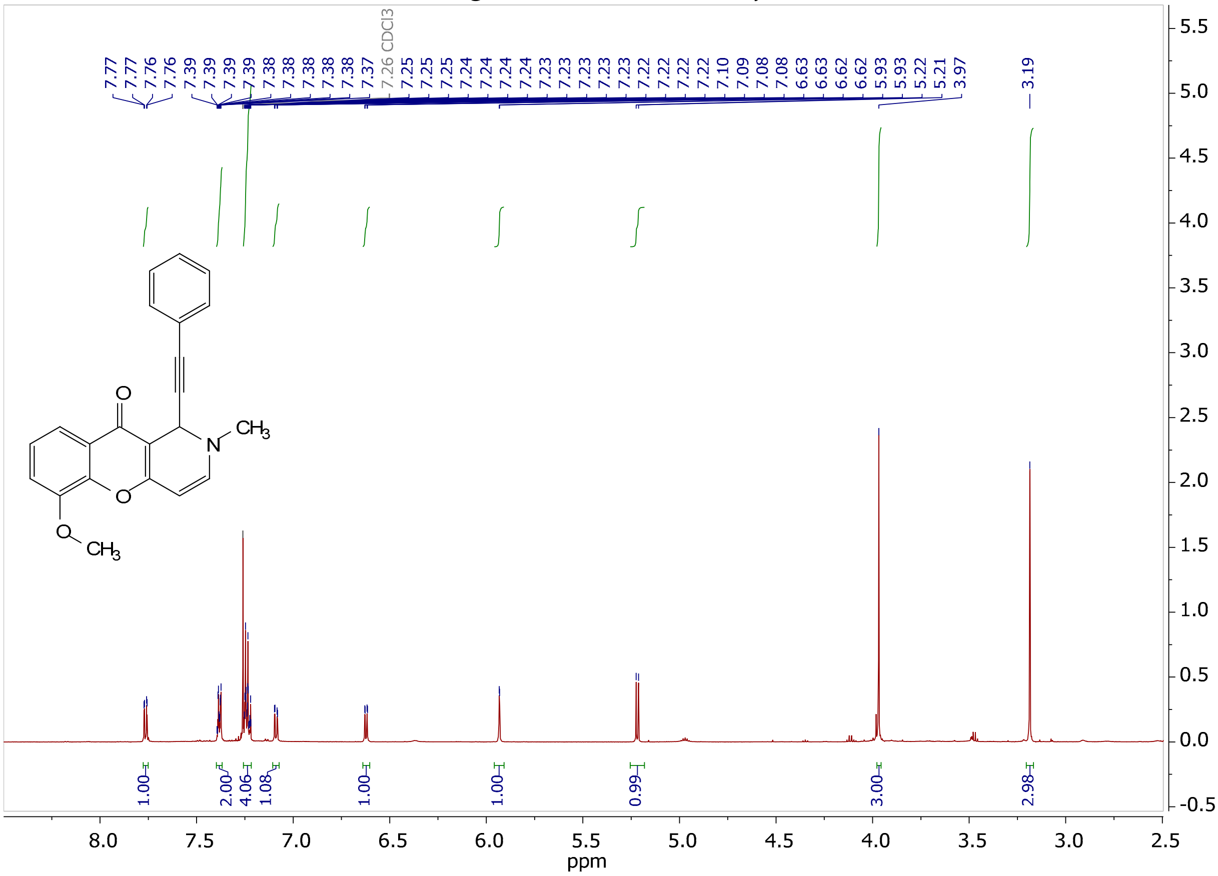

Figure S7. ^{13}C *NMR data of* ***3b***

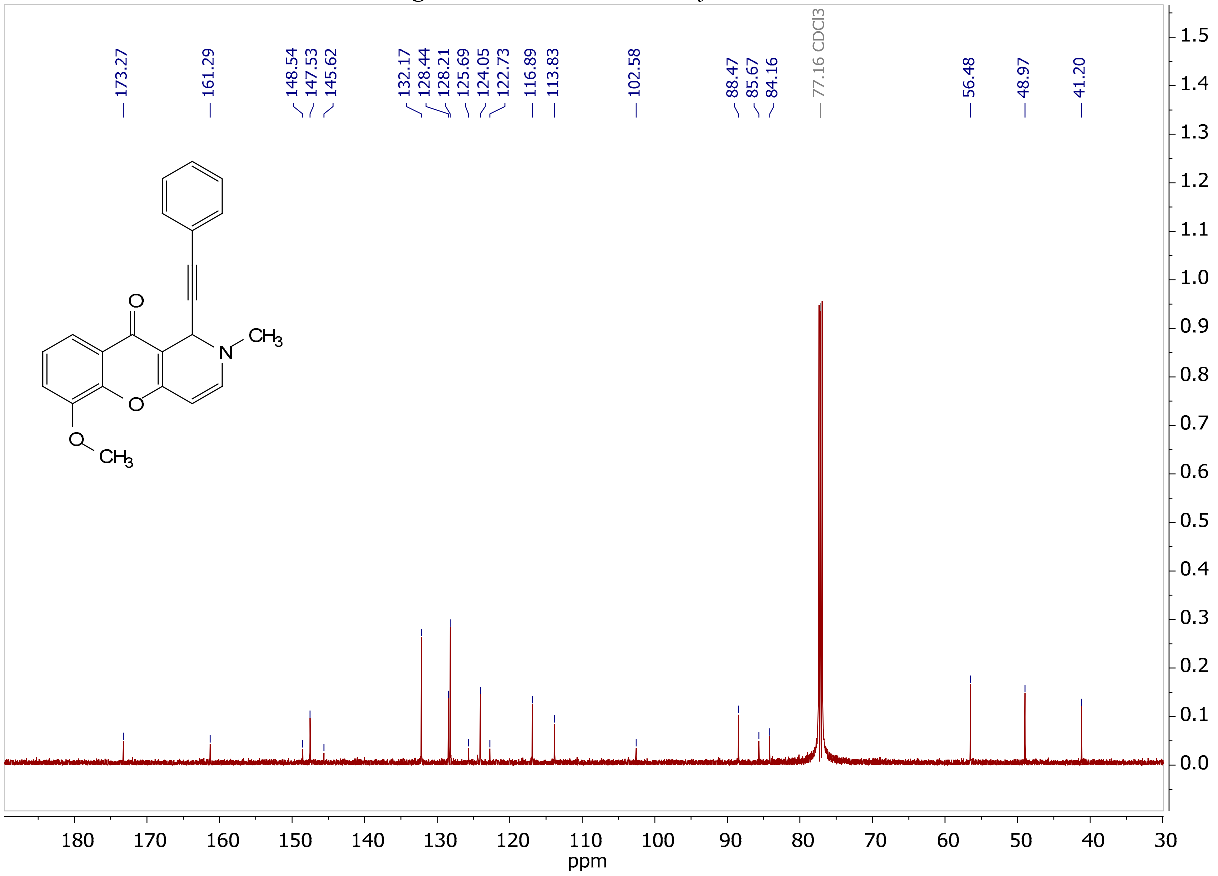

Figure S8. ^{1}H *NMR data of* ***3c***

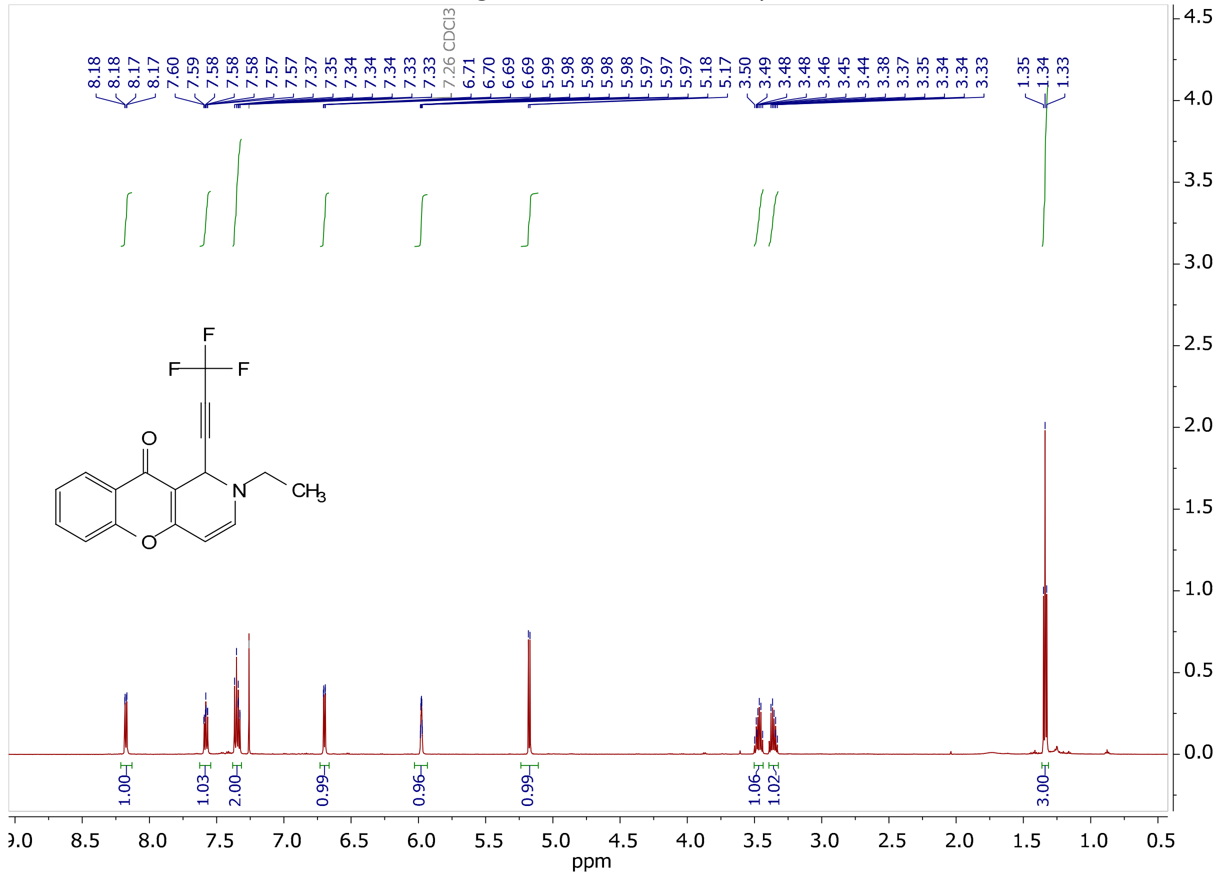

Figure S9. ^{13}C NMR data of **3c**

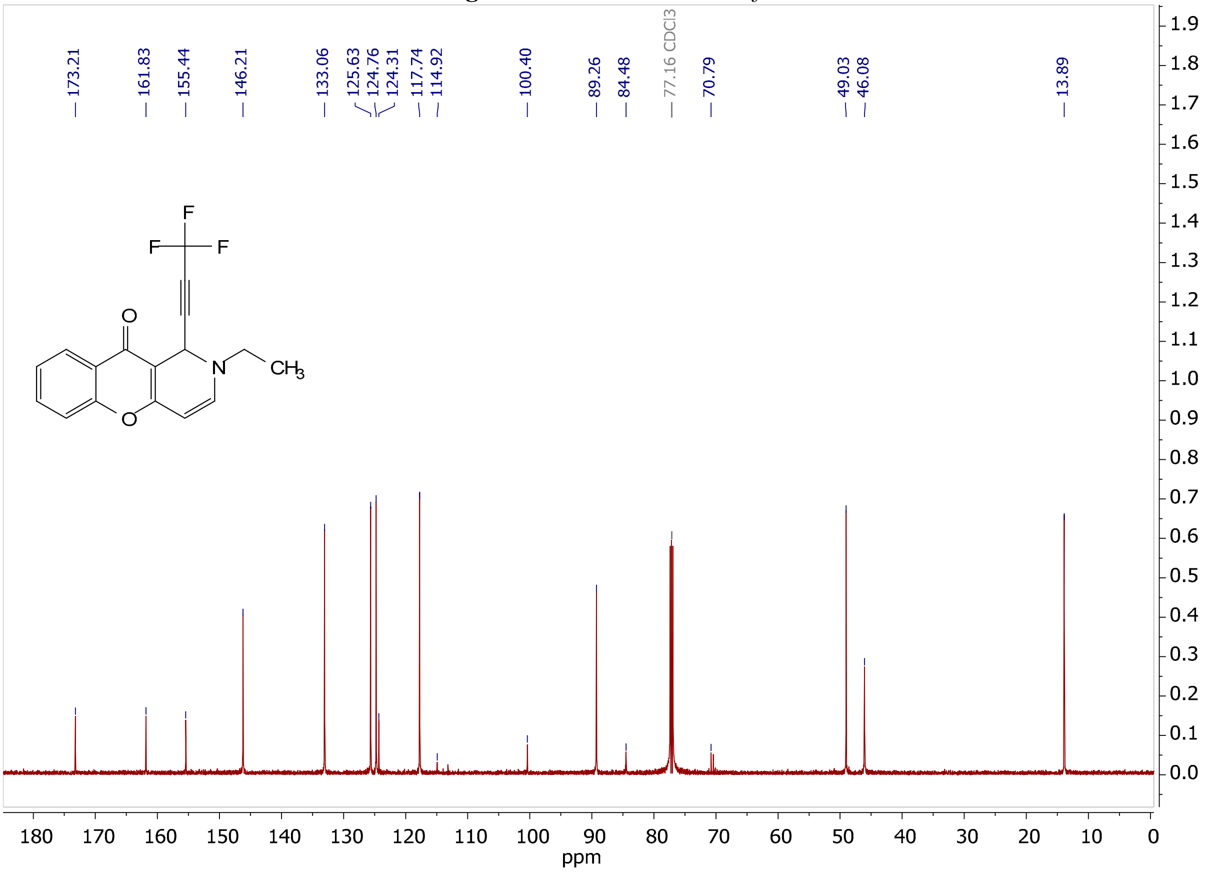

Figure S10. ^{13}C NMR data of **3d**

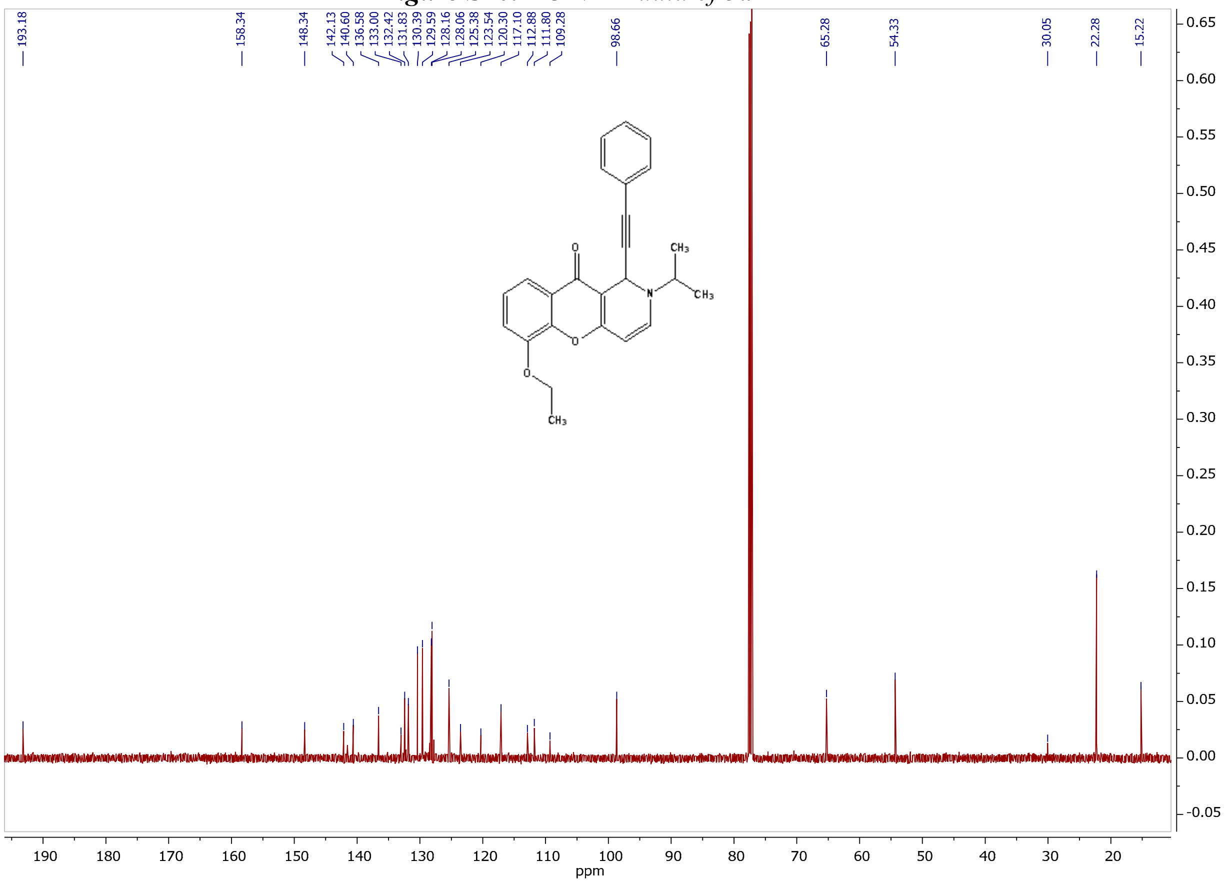

Figure S11. ¹H NMR data of **4a**

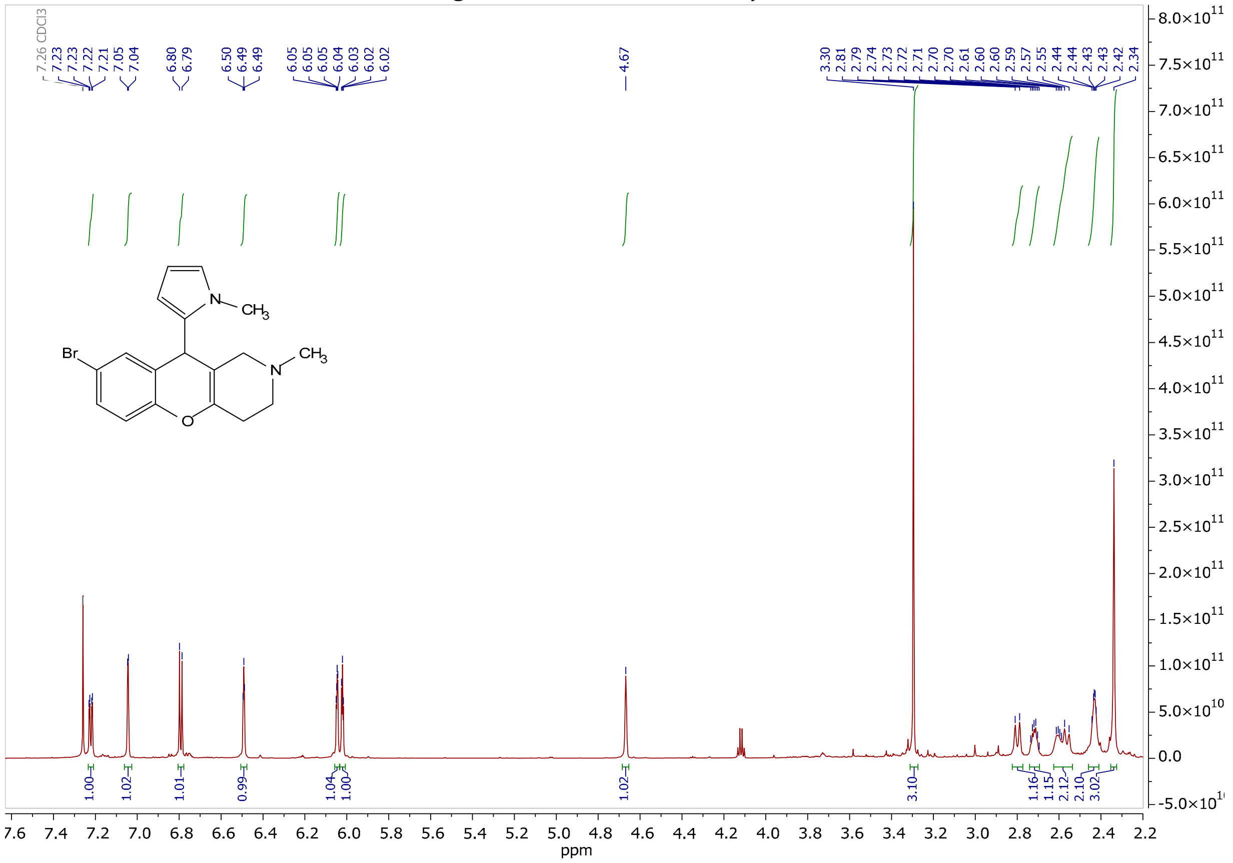

Figure S12. ¹H NMR data of **5**

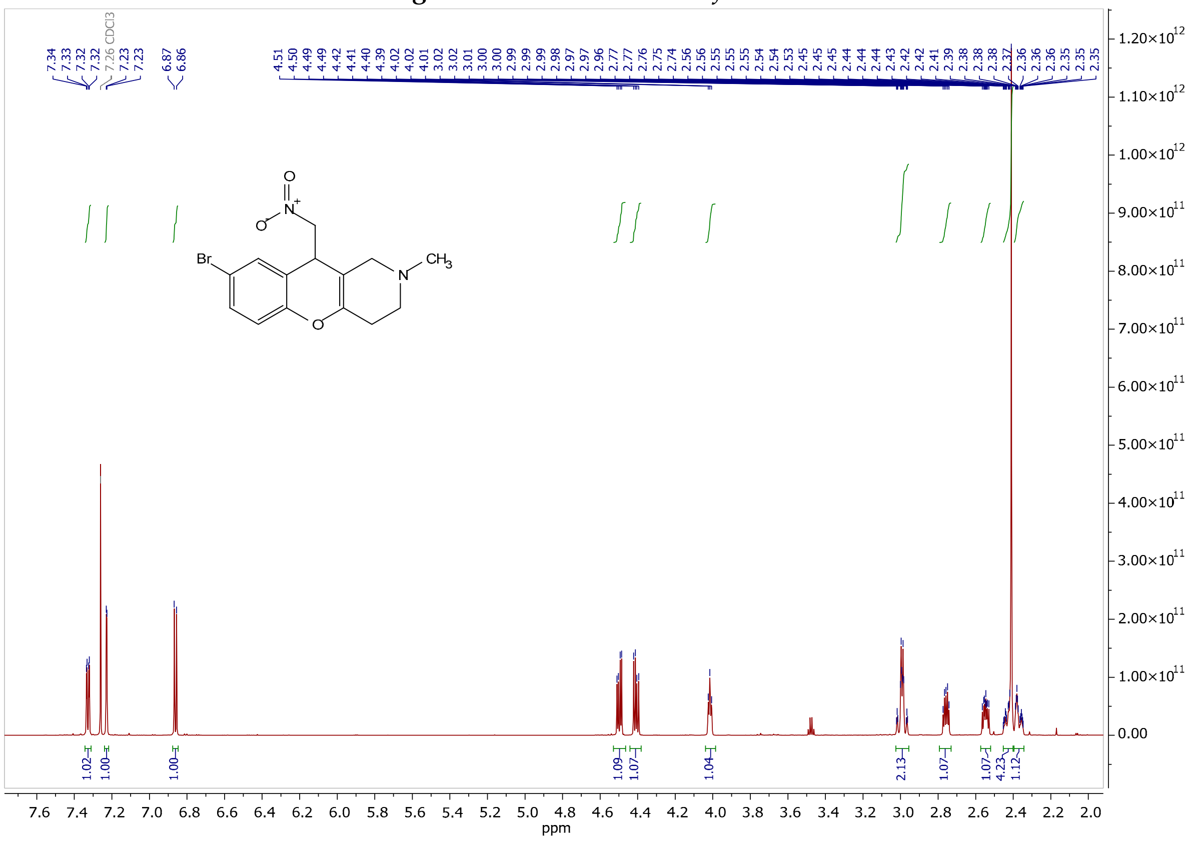

Figure S13. *^{13}C NMR data of* ***5***

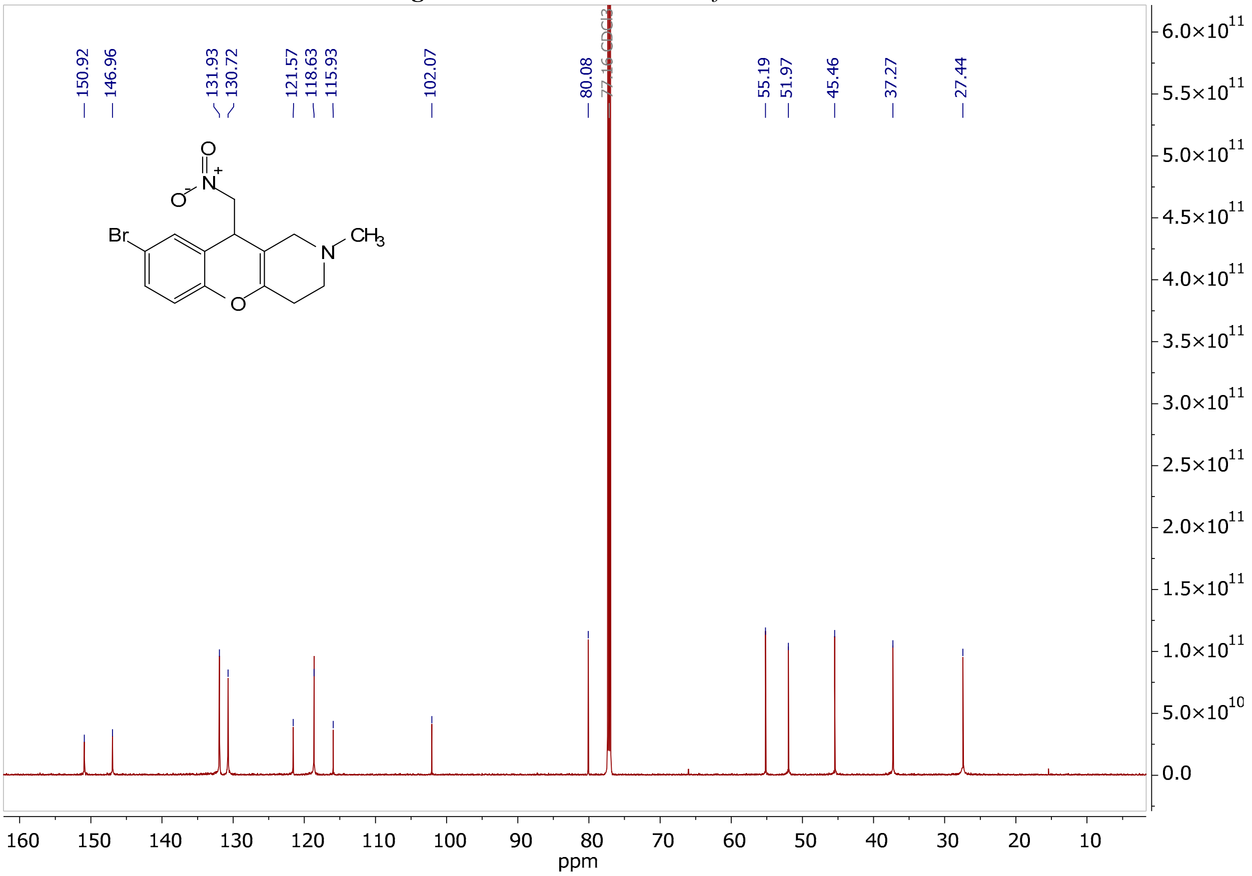

Figure S14*. ^{1}H NMR data of* ***6a***

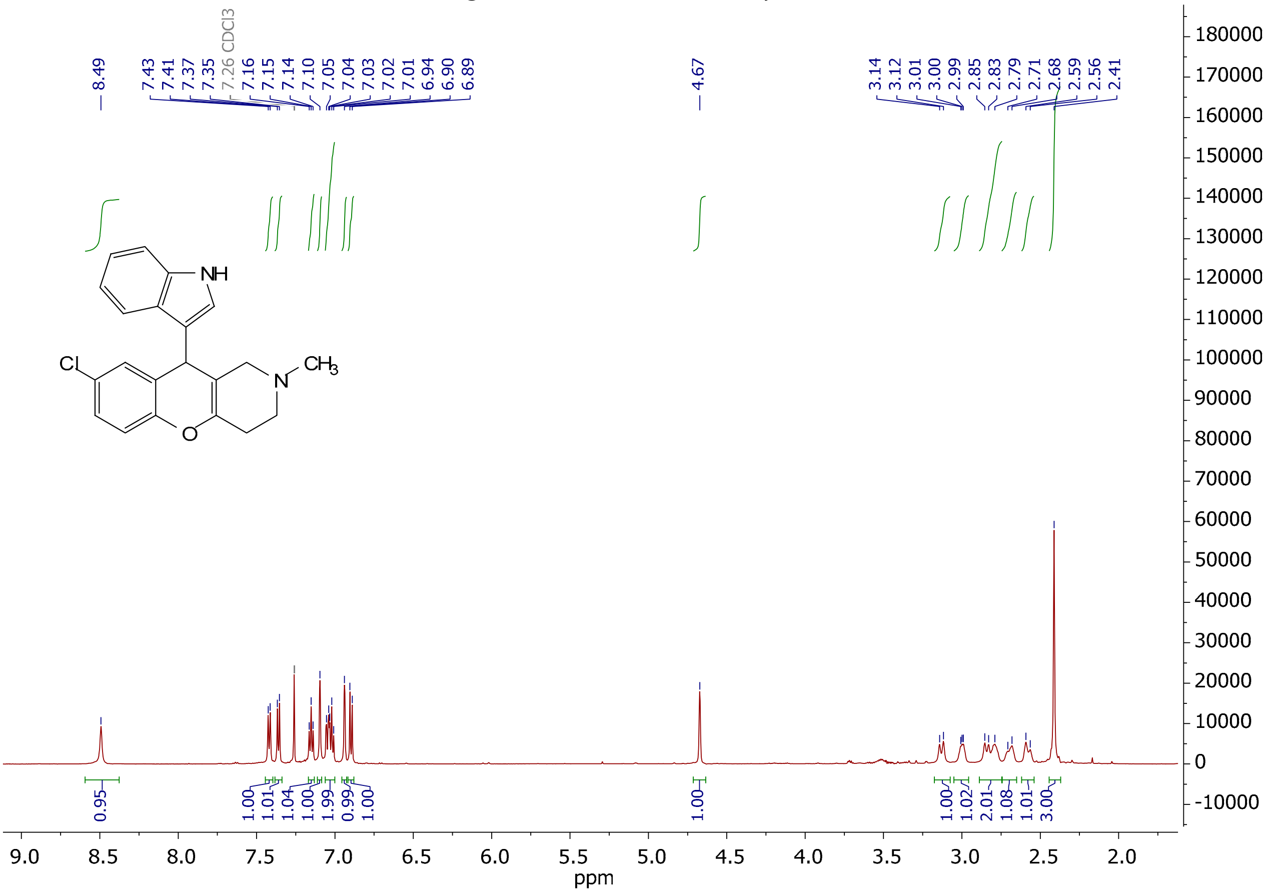

Figure S15. *^{13}C NMR data of* ***6a***

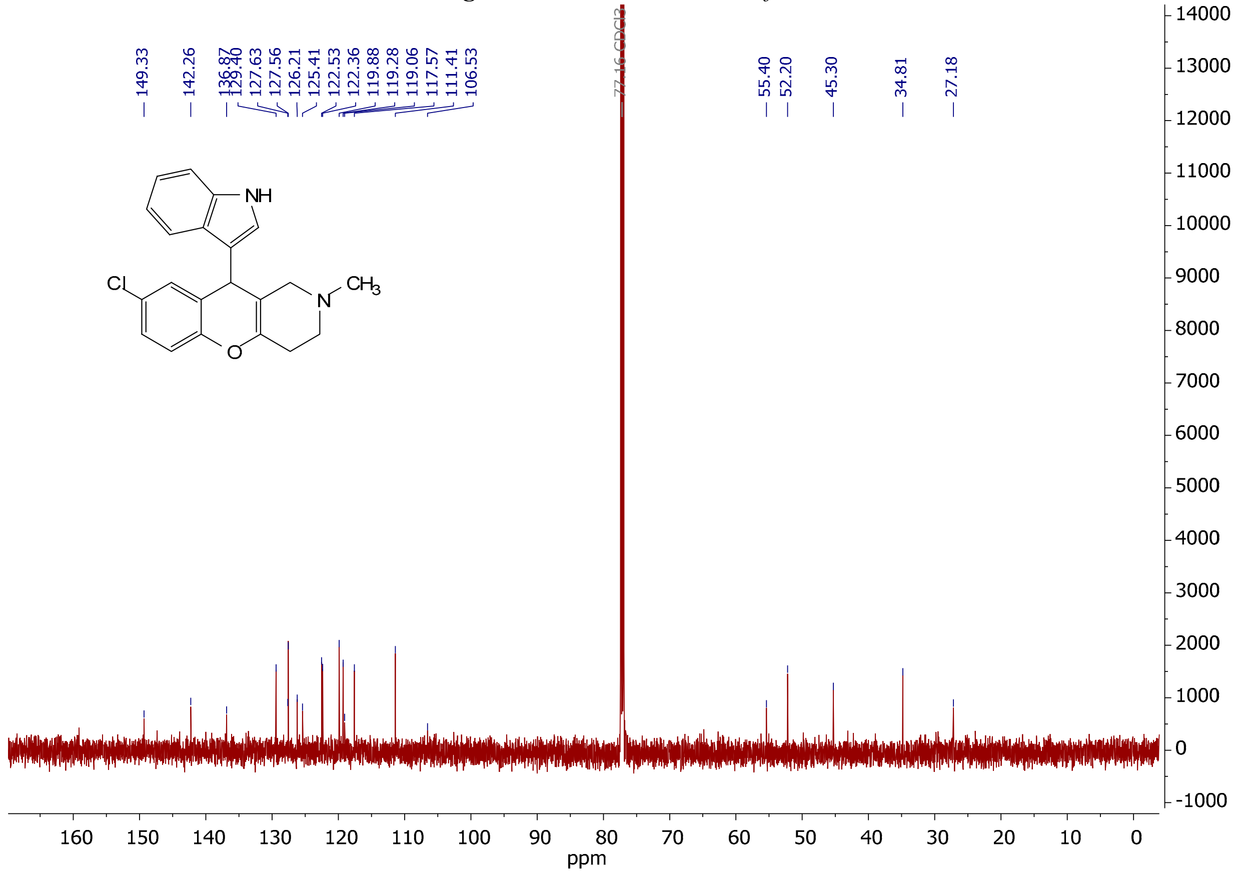

Figure S16*. ^{1}H NMR data of* ***6b***

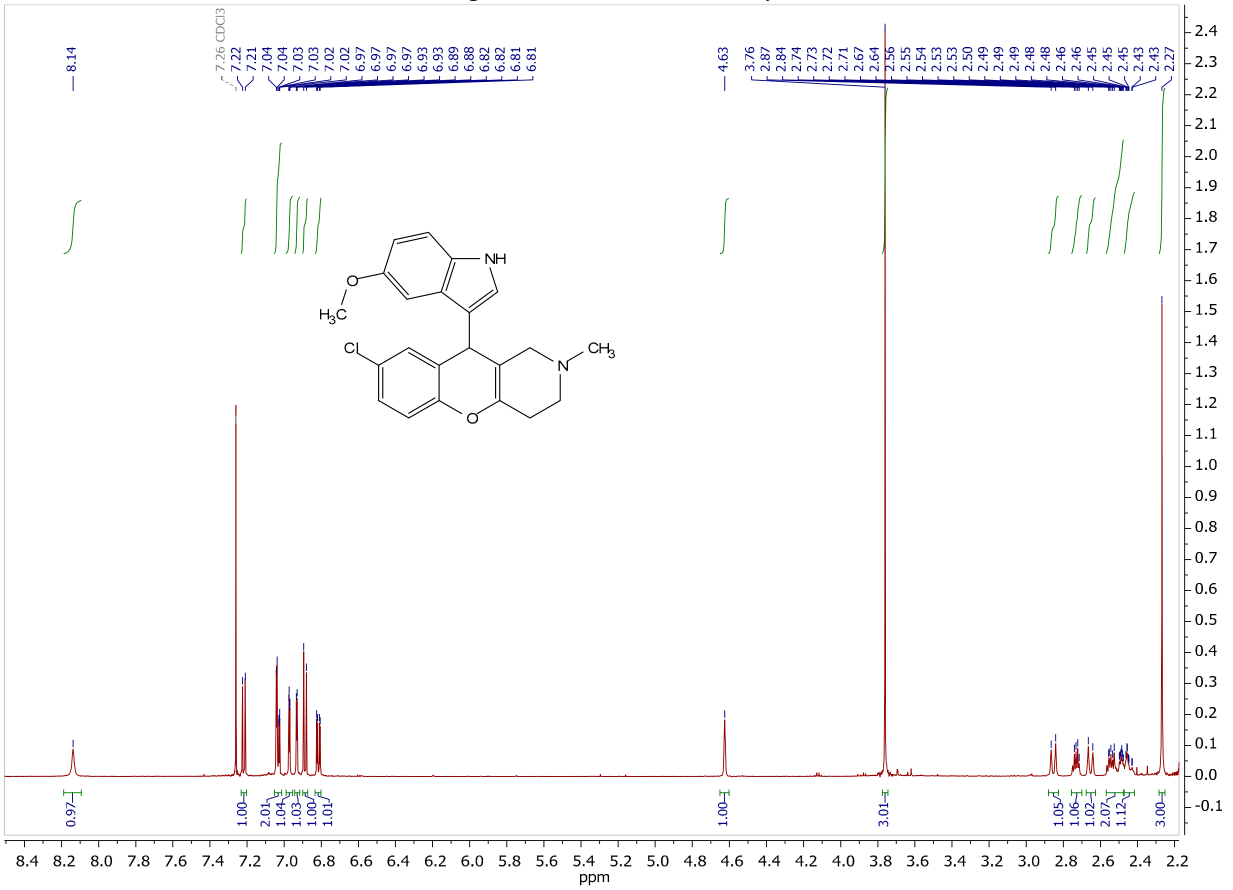

Figure S17. *^{13}C NMR data of* ***6b***

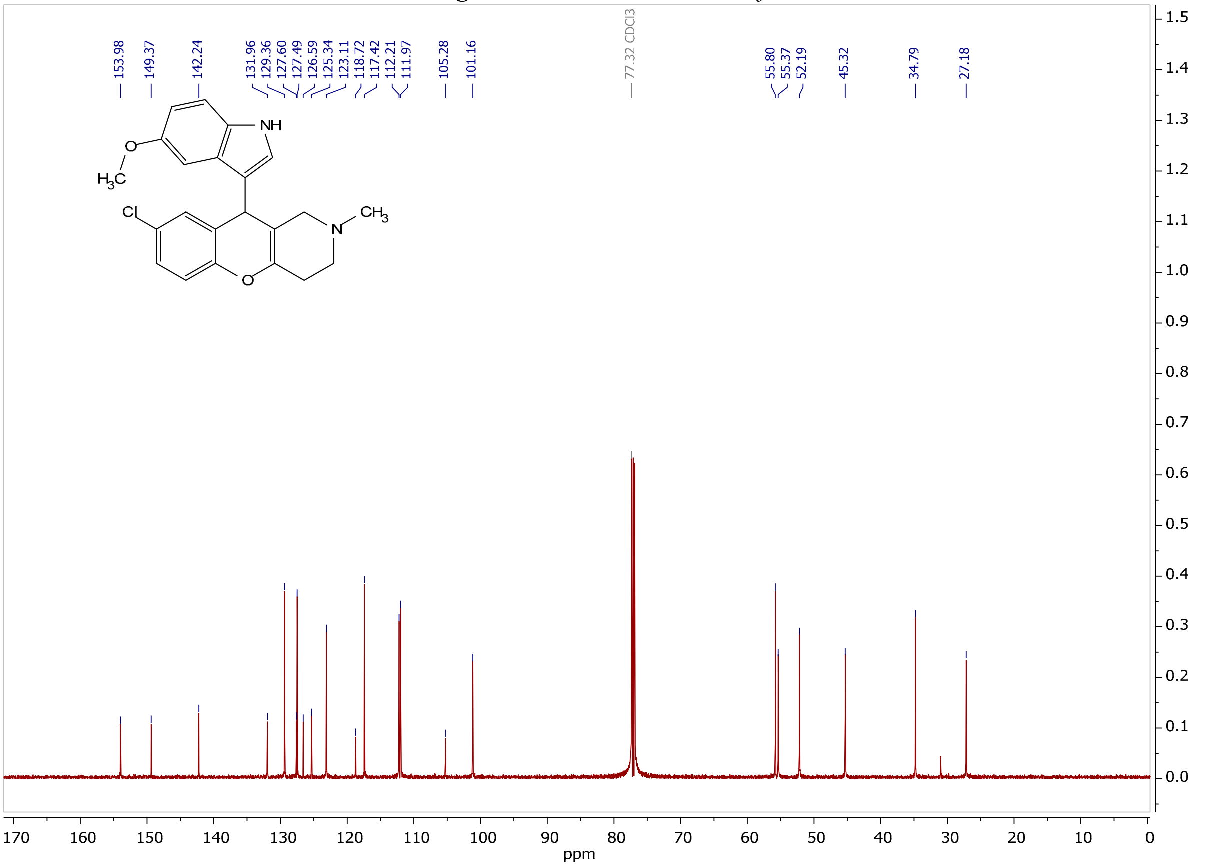

Figure S18*.* *^{1}H NMR data of* ***6c***

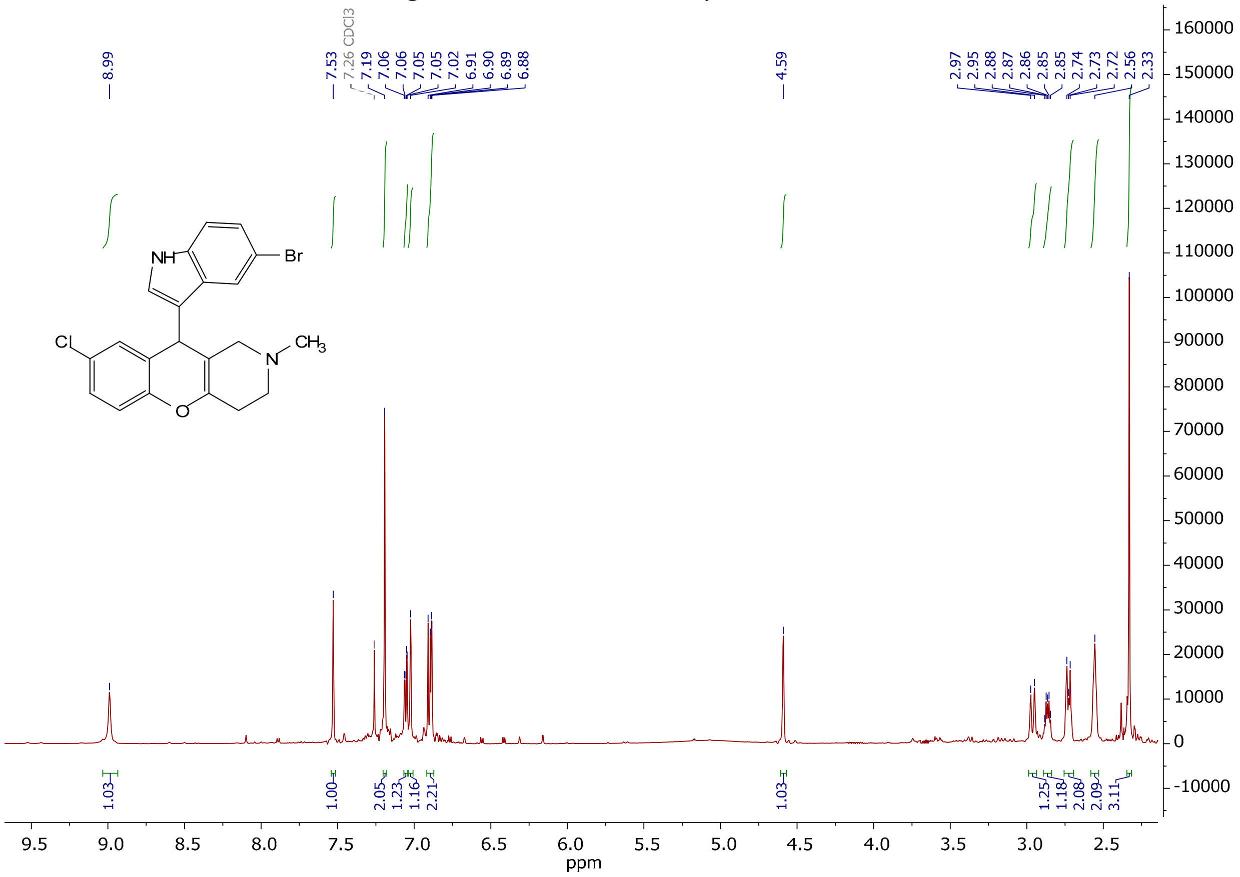

Figure S19. ^{1}H NMR data of ***7a***

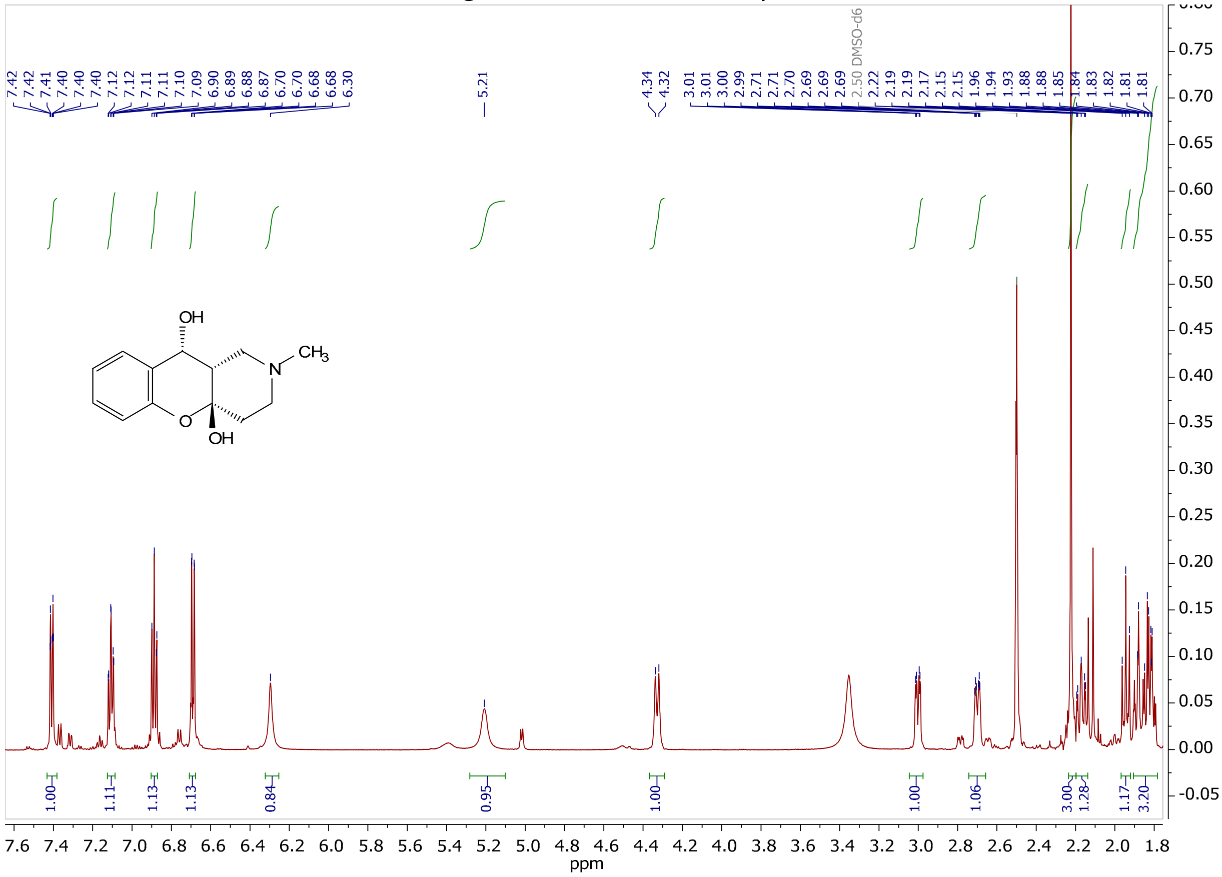

Figure S20. ^{13}C NMR data of ***7a***

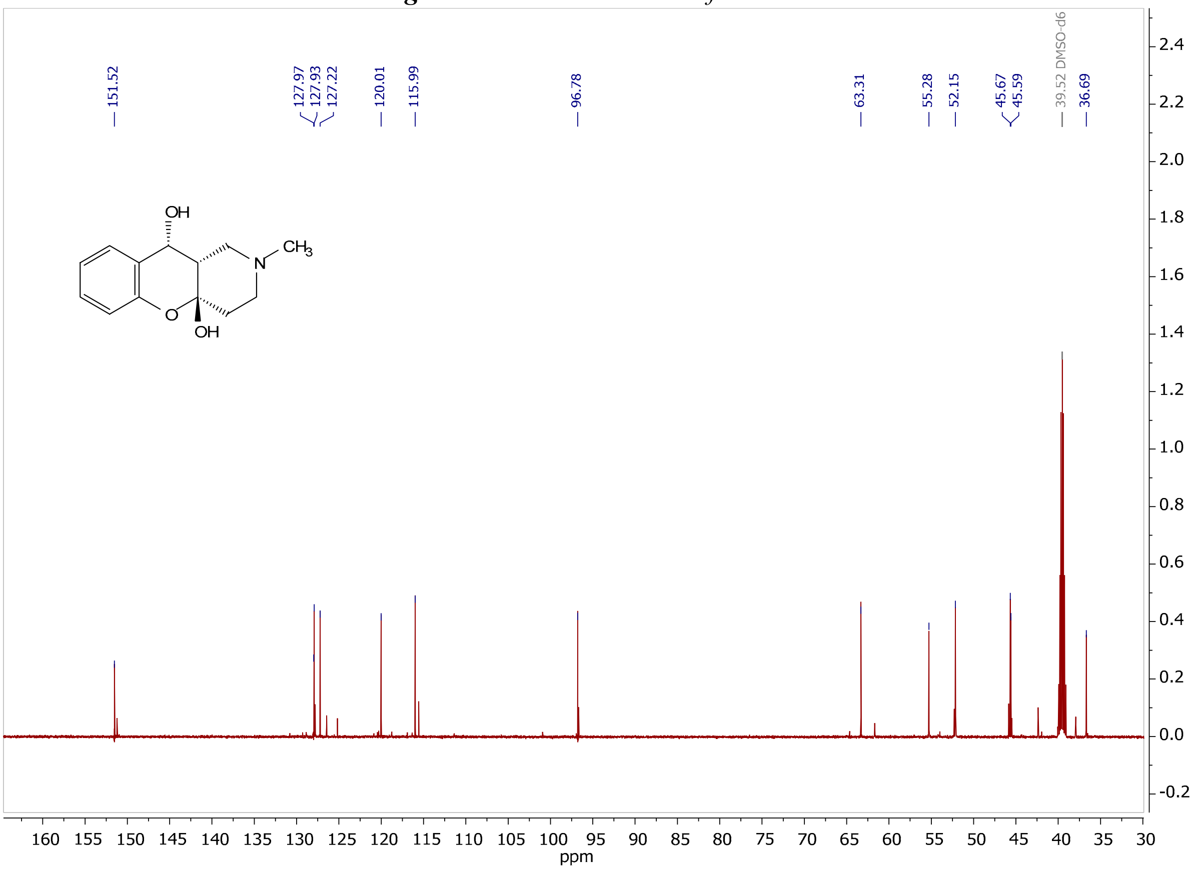

Figure S21. ^{1}H NMR data of ***7b***

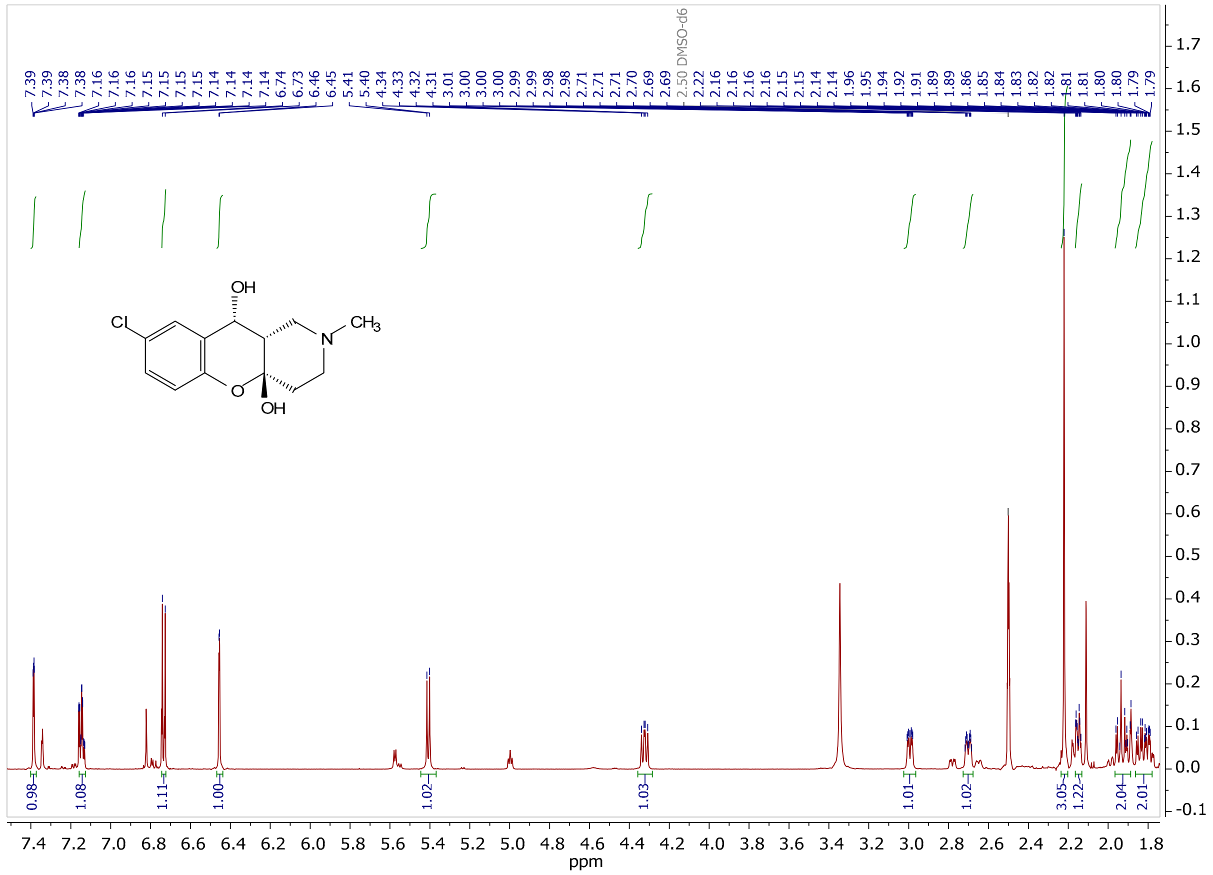

Figure S22. ^{13}C NMR data of ***7b***

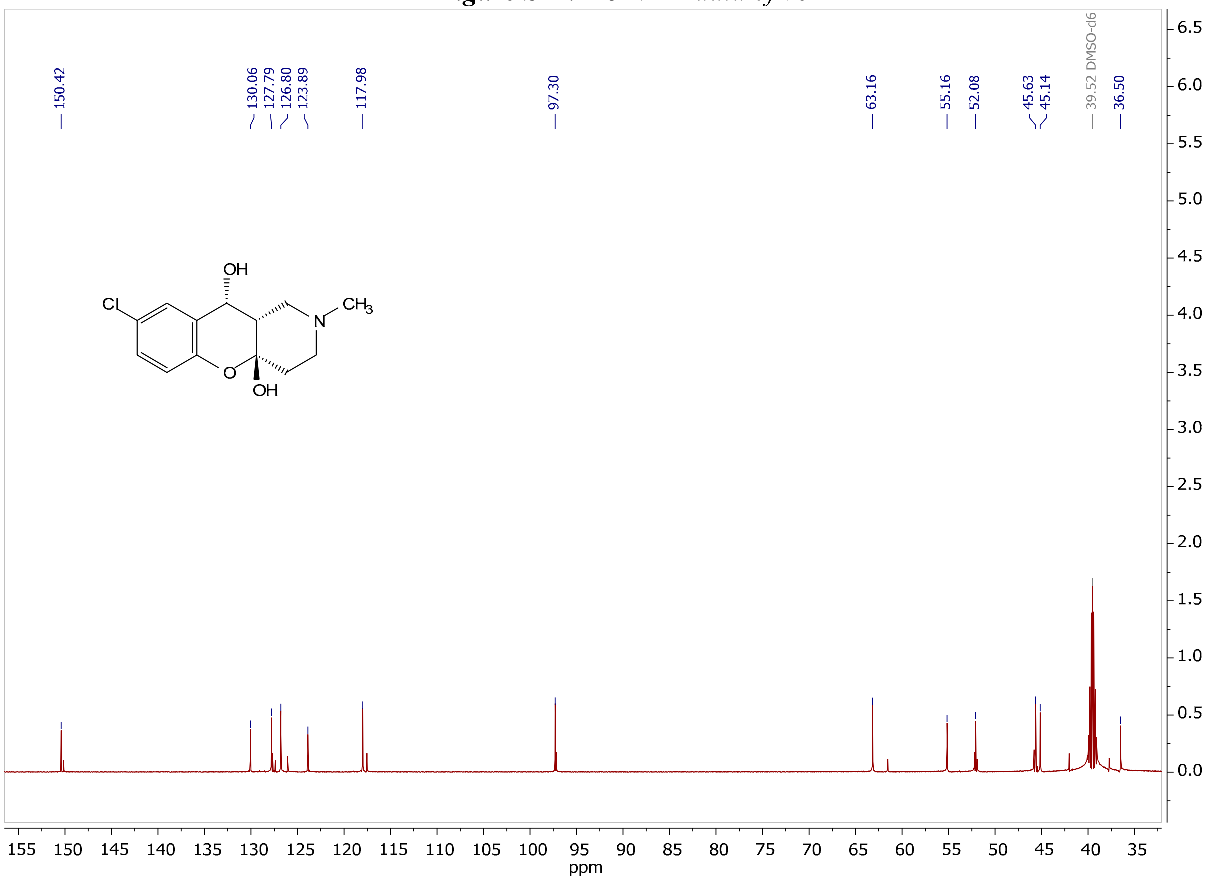

Figure S23. 1H NMR data of **7c**

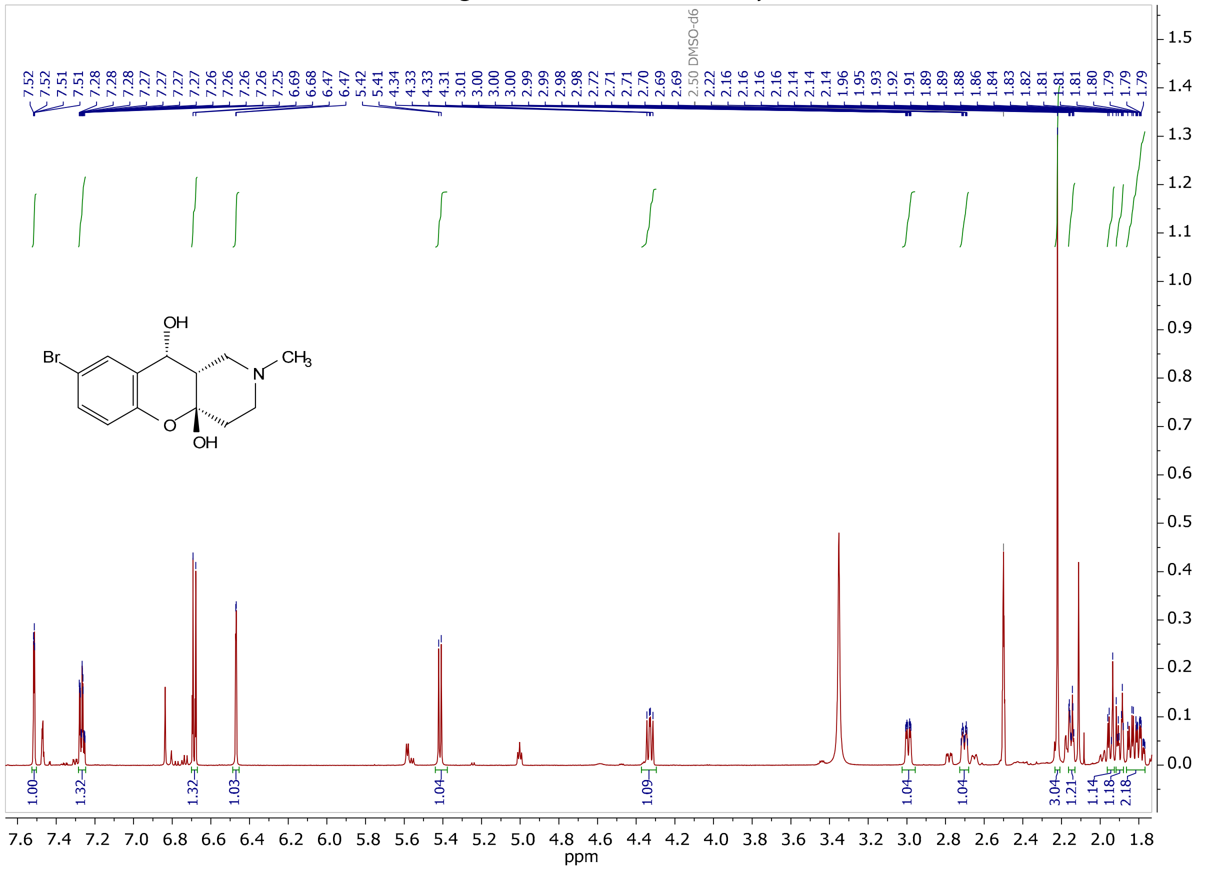

Figure S24. 13C NMR data of *7c*

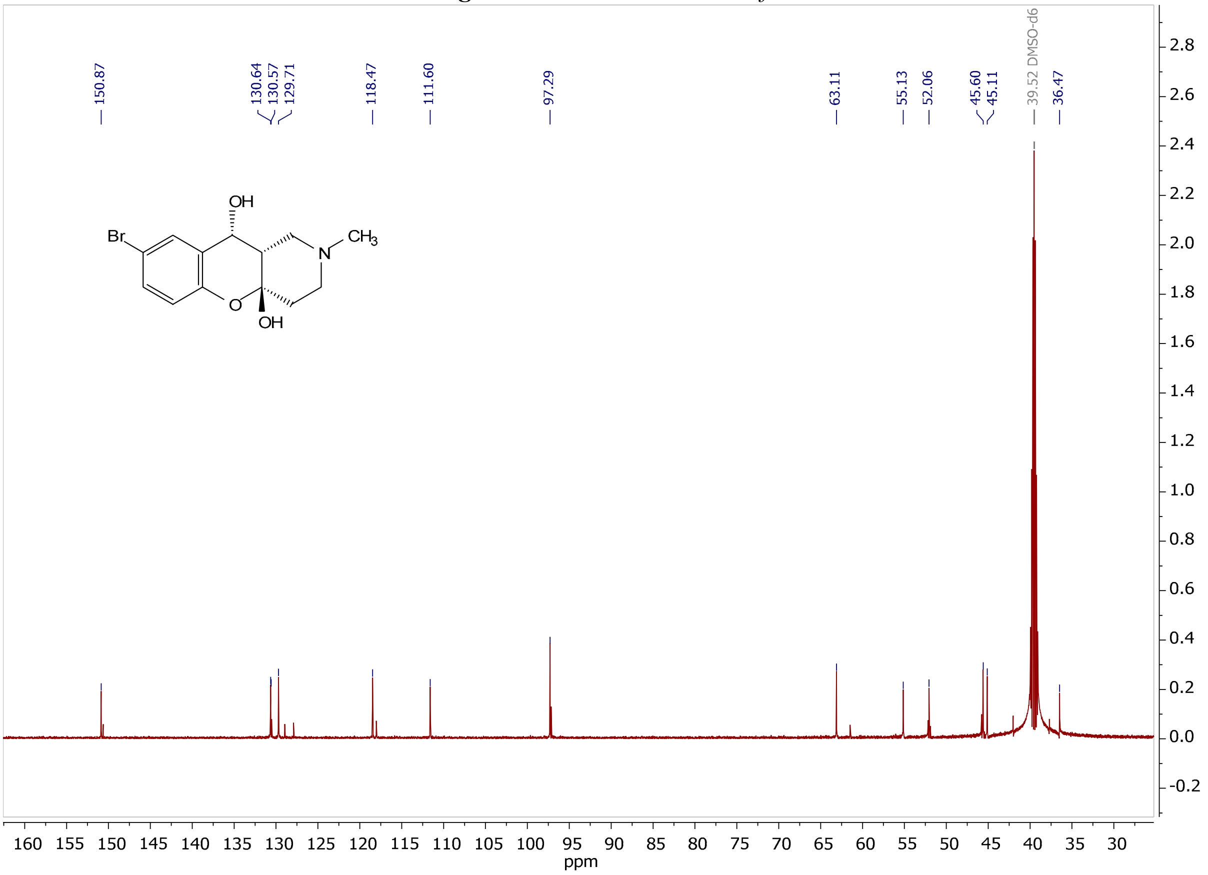

Figure S25. ¹H NMR data of ***7d***

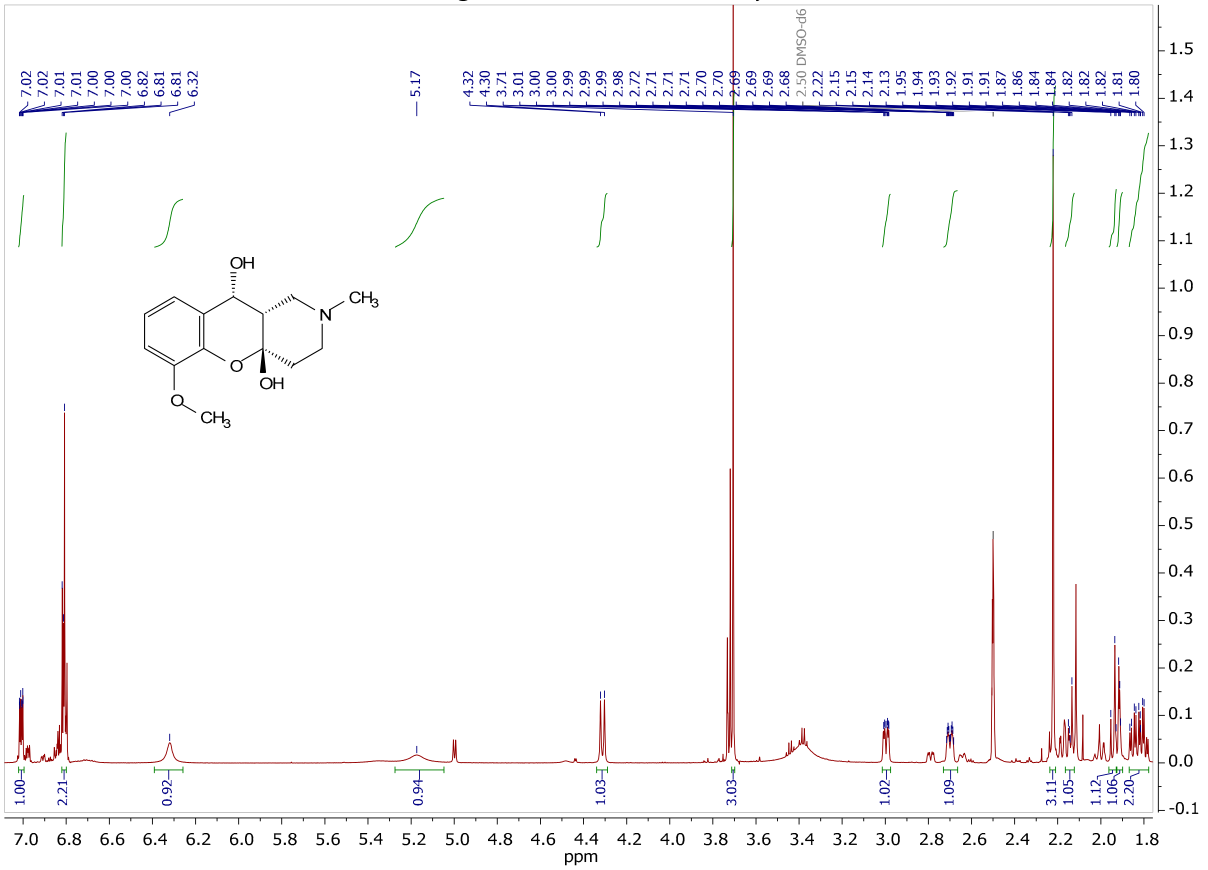

Figure S26. ¹³C NMR data of ***7d***

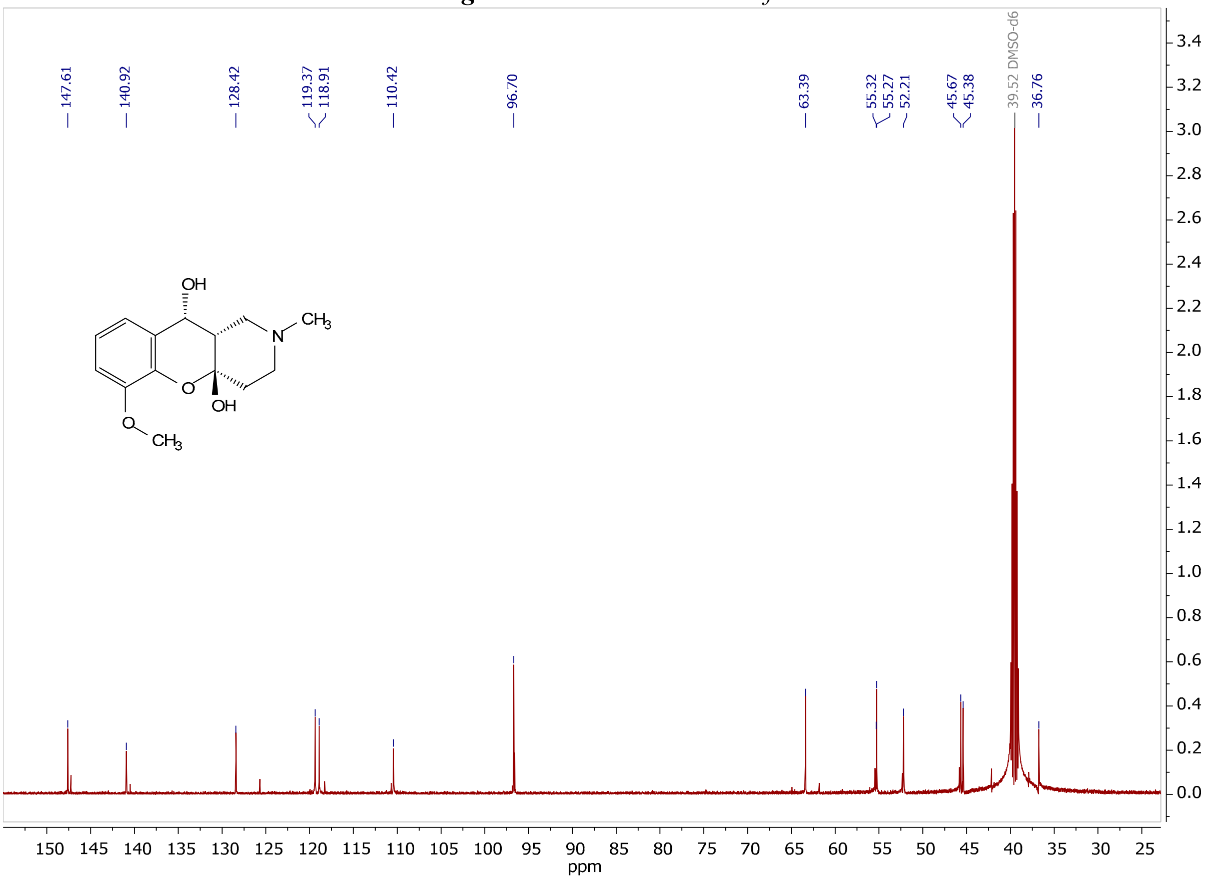

Figure S27. ^{1}H NMR data of **7e**

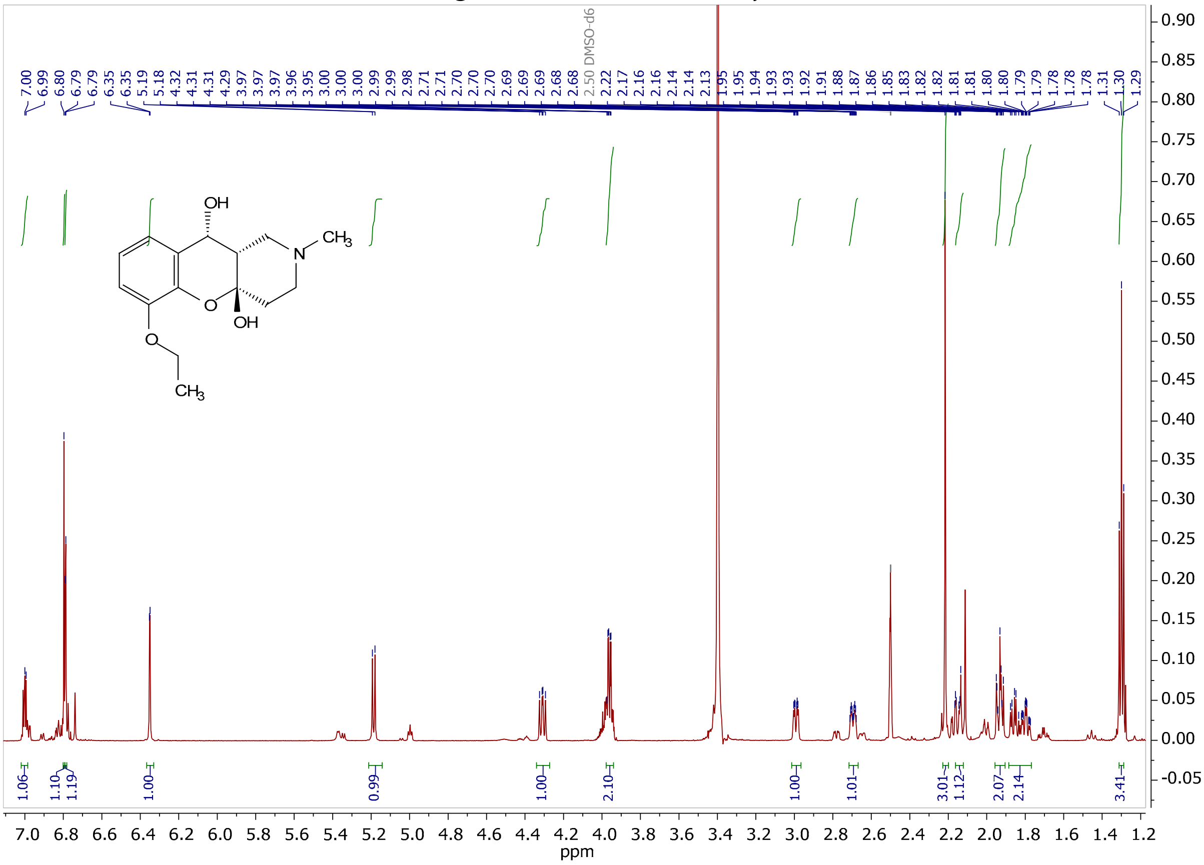

Figure S28. ^{13}C NMR data of **7e**

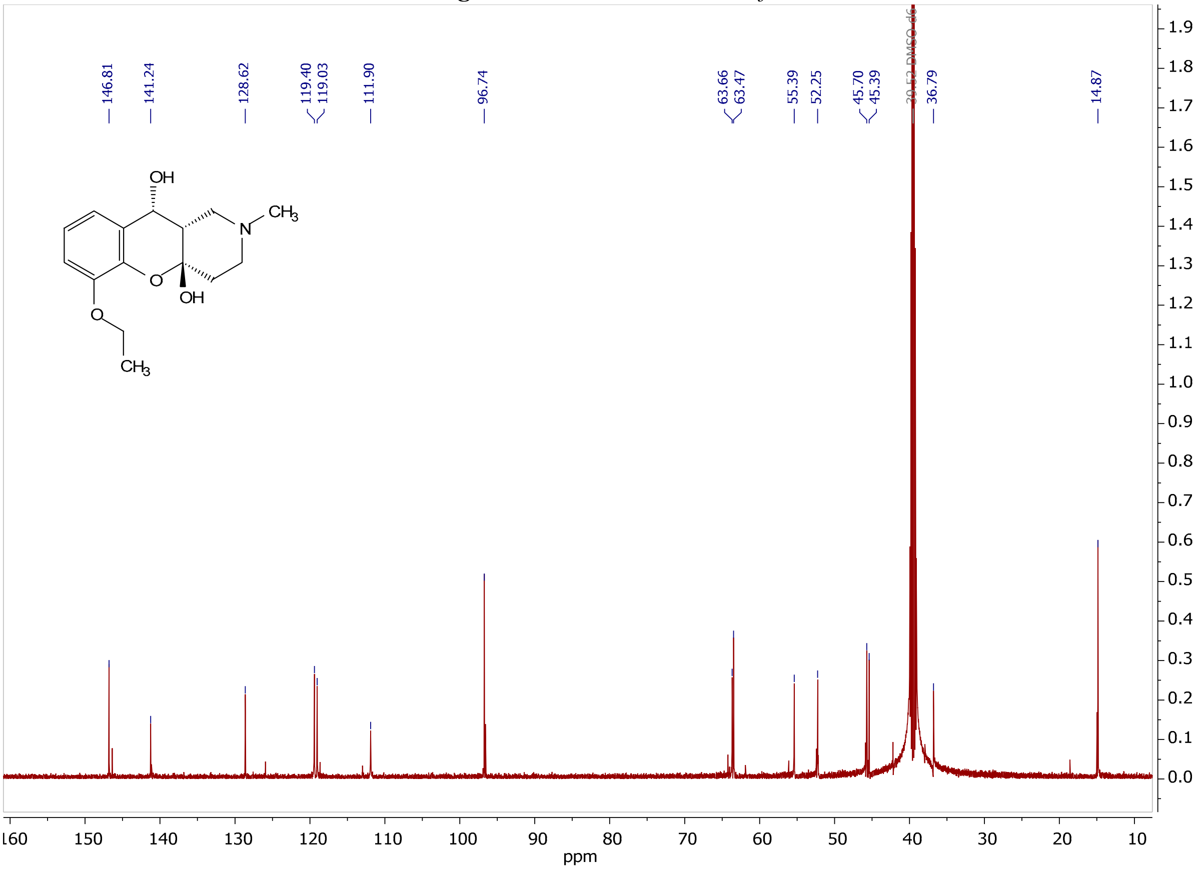

Figure S29. ^{1}H NMR data of ***7f***

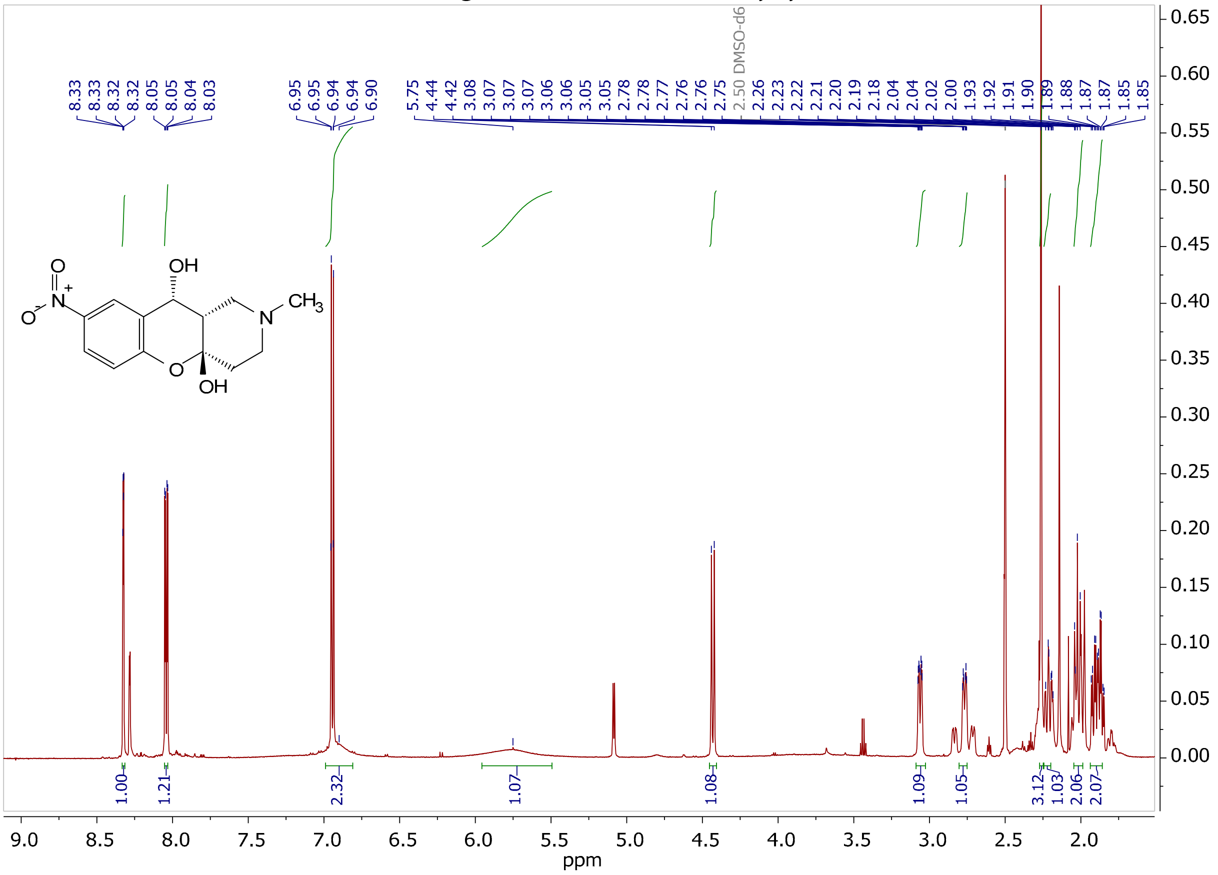

Figure S30. ^{13}C NMR data of ***7f***

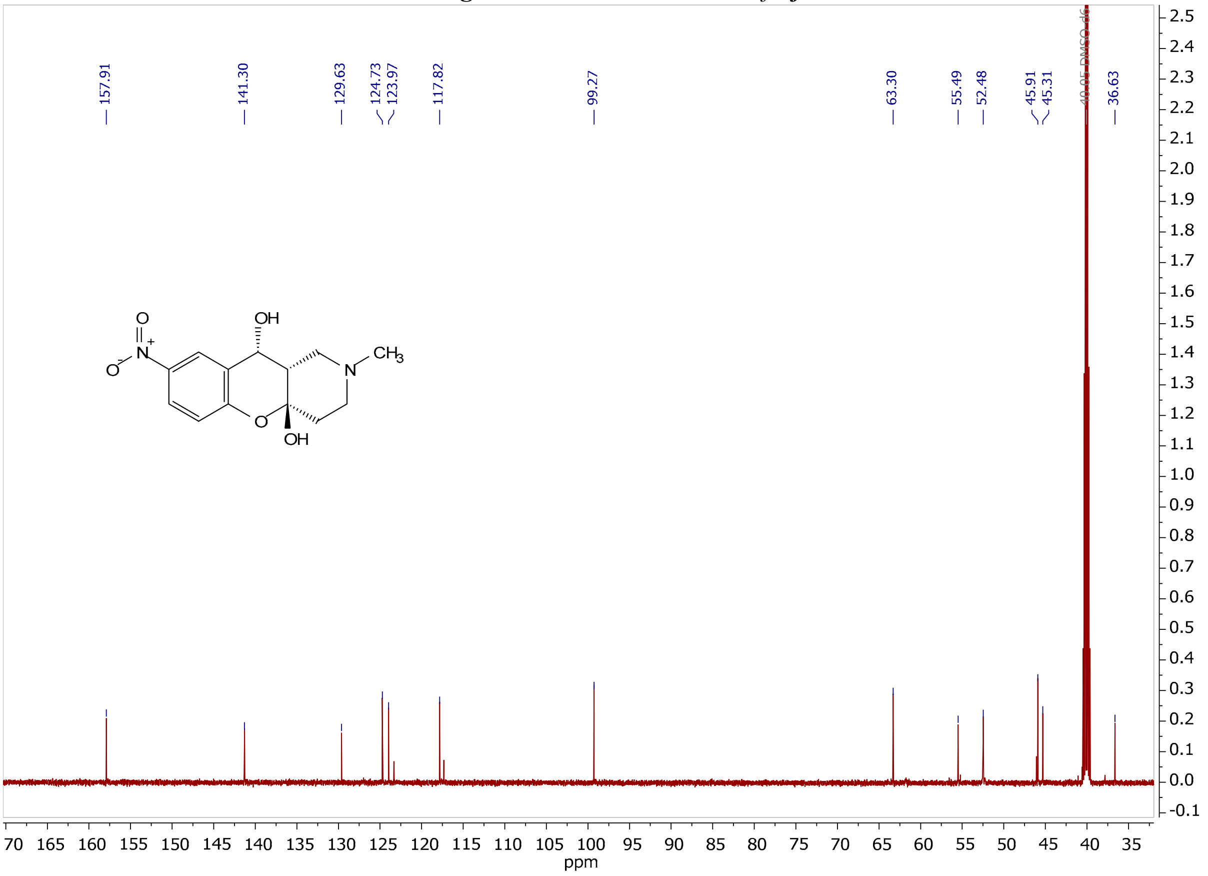

Figure S31. ¹H NMR data of ***8a***

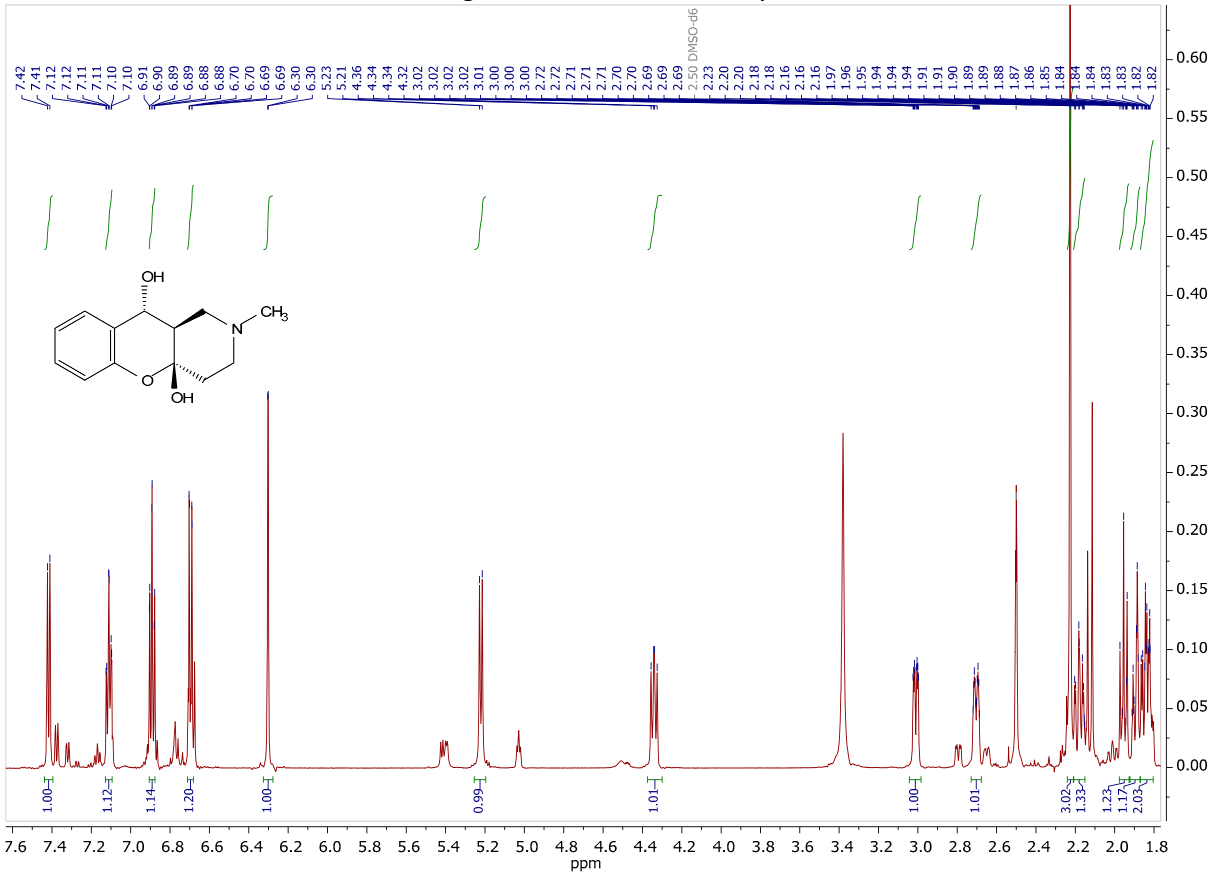

Figure S32. ^{13}*C NMR data of* ***8a***

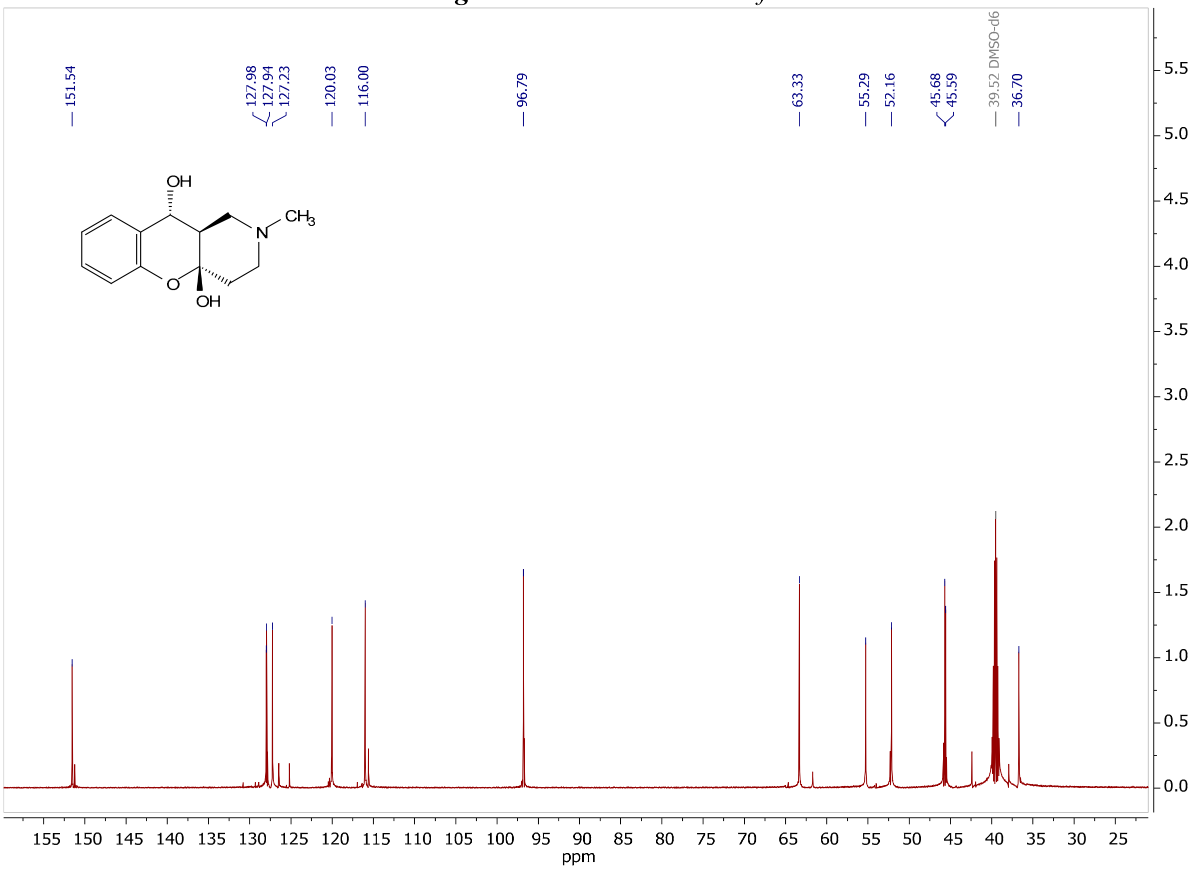

Figure S33. ^{1}H NMR data of **8b**

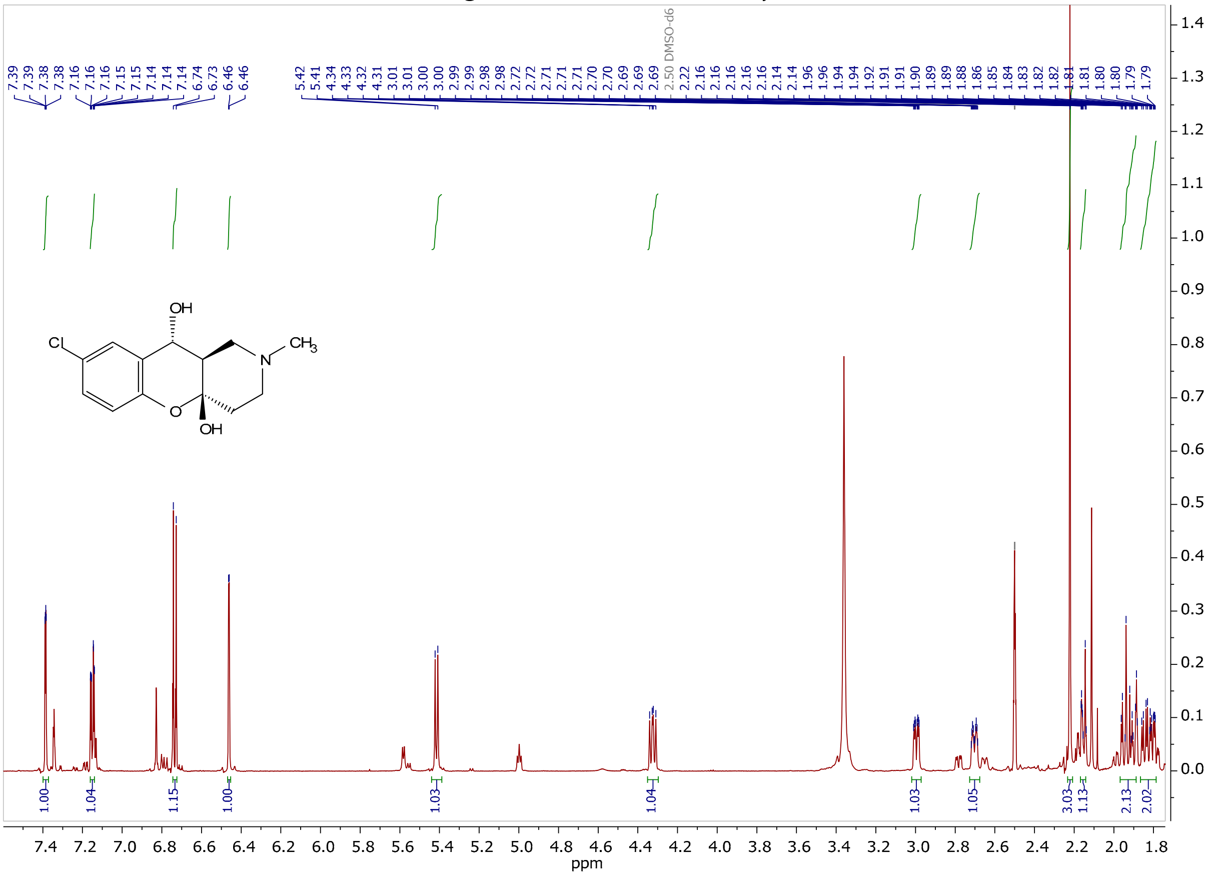

Figure S34. ^{13}C NMR data of **8b**

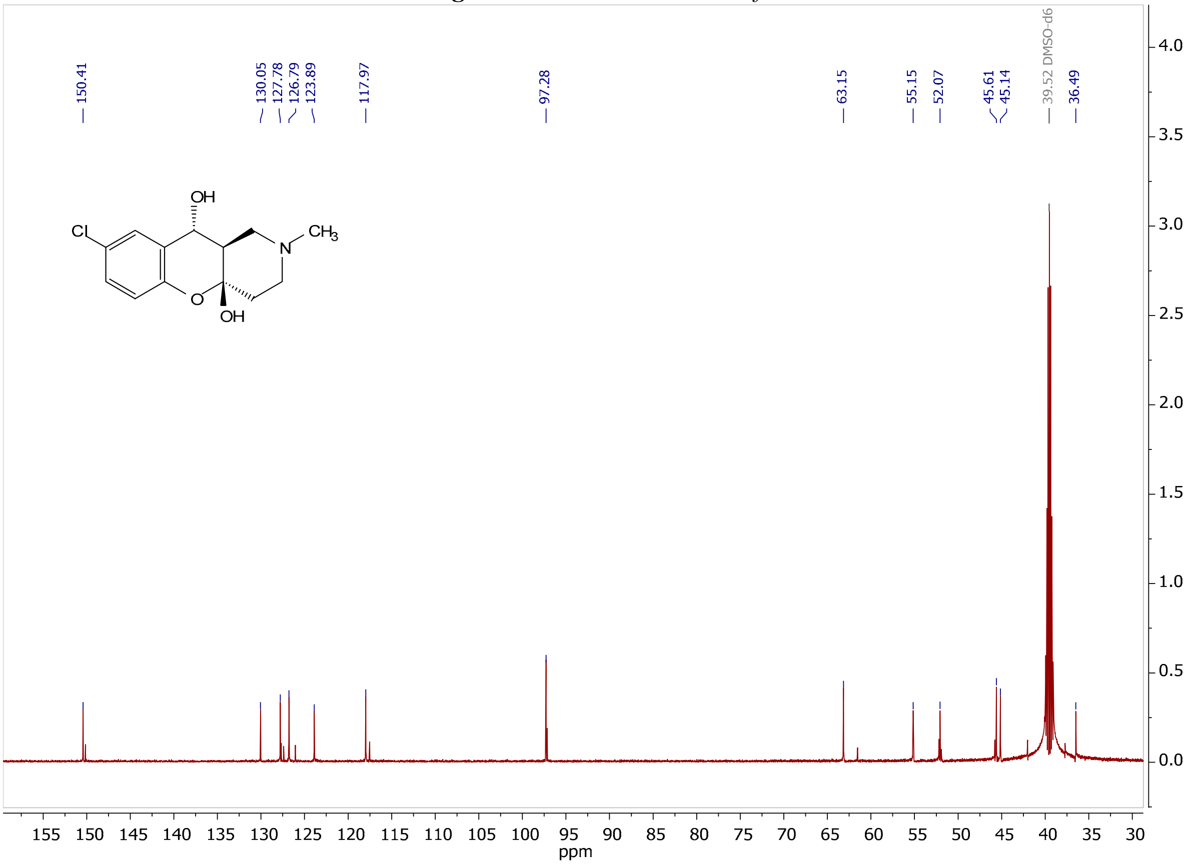

Figure S35. ^{1}H NMR data of ***8c***

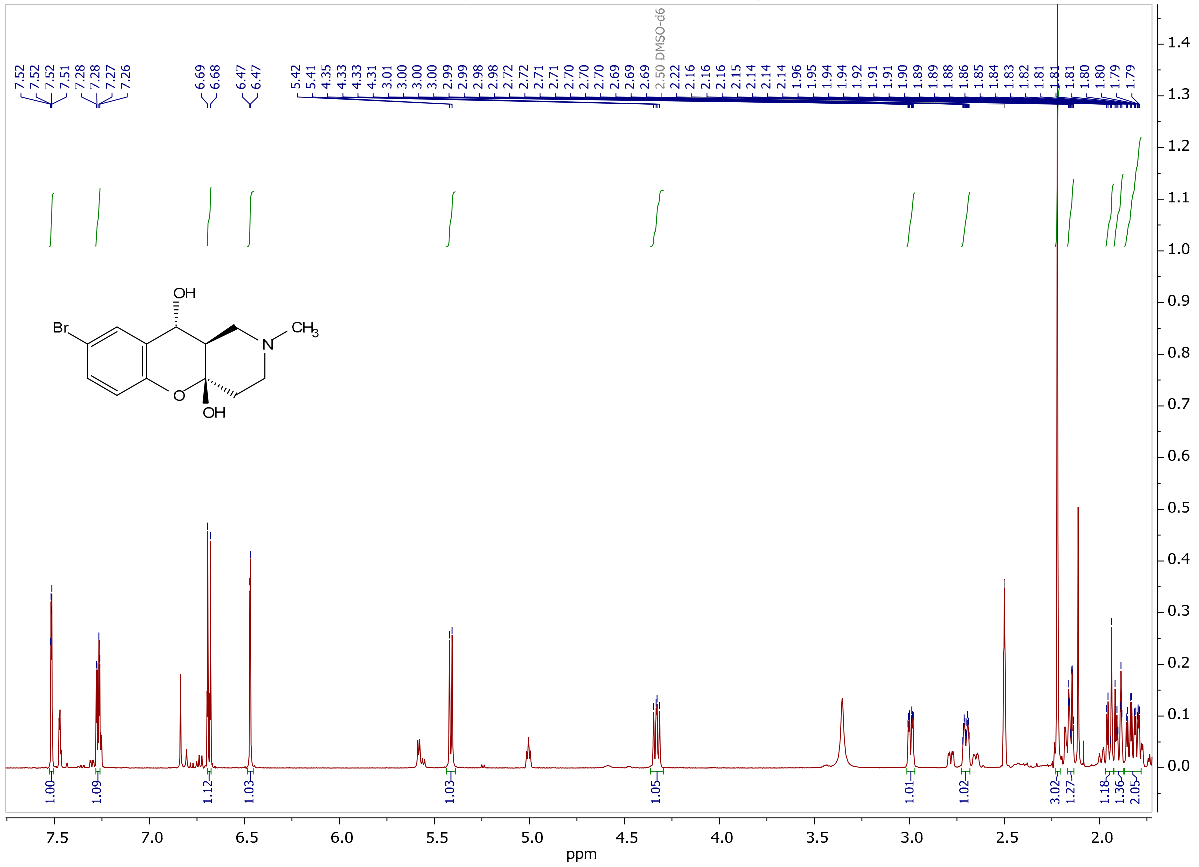

Figure S36. ^{13}C NMR data of ***8c***

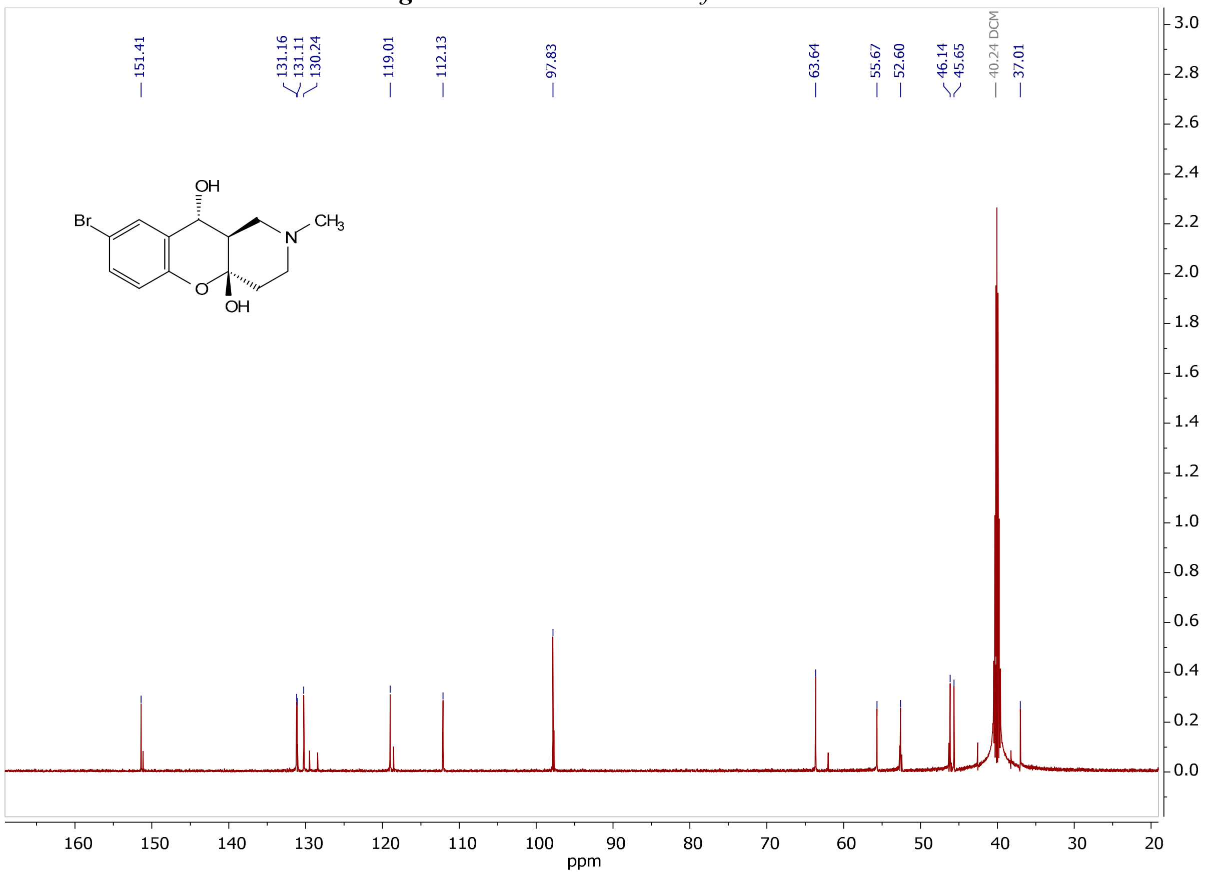

Figure S37. ¹H NMR data of ***8d***

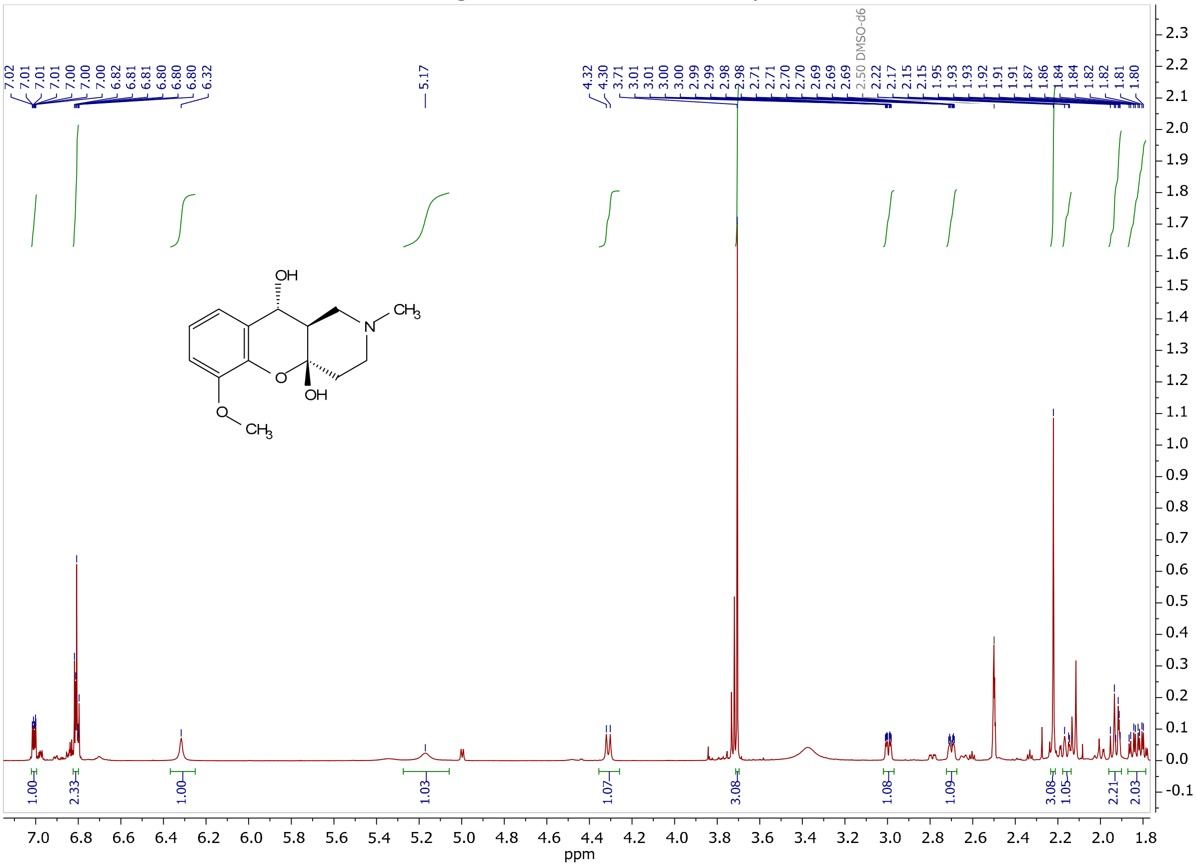

Figure S38. ^{13}C NMR data of ***8d***

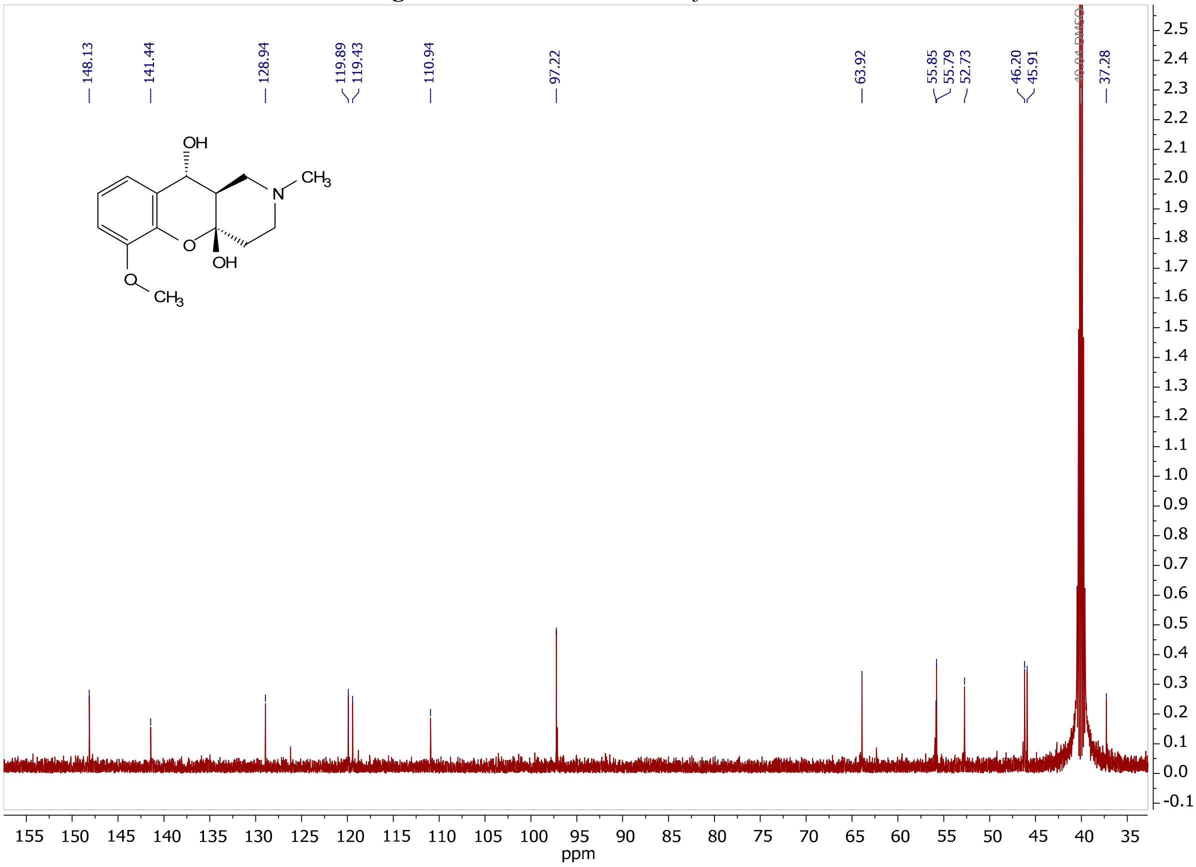

Figure 39. ^{1}H NMR data of **8e**

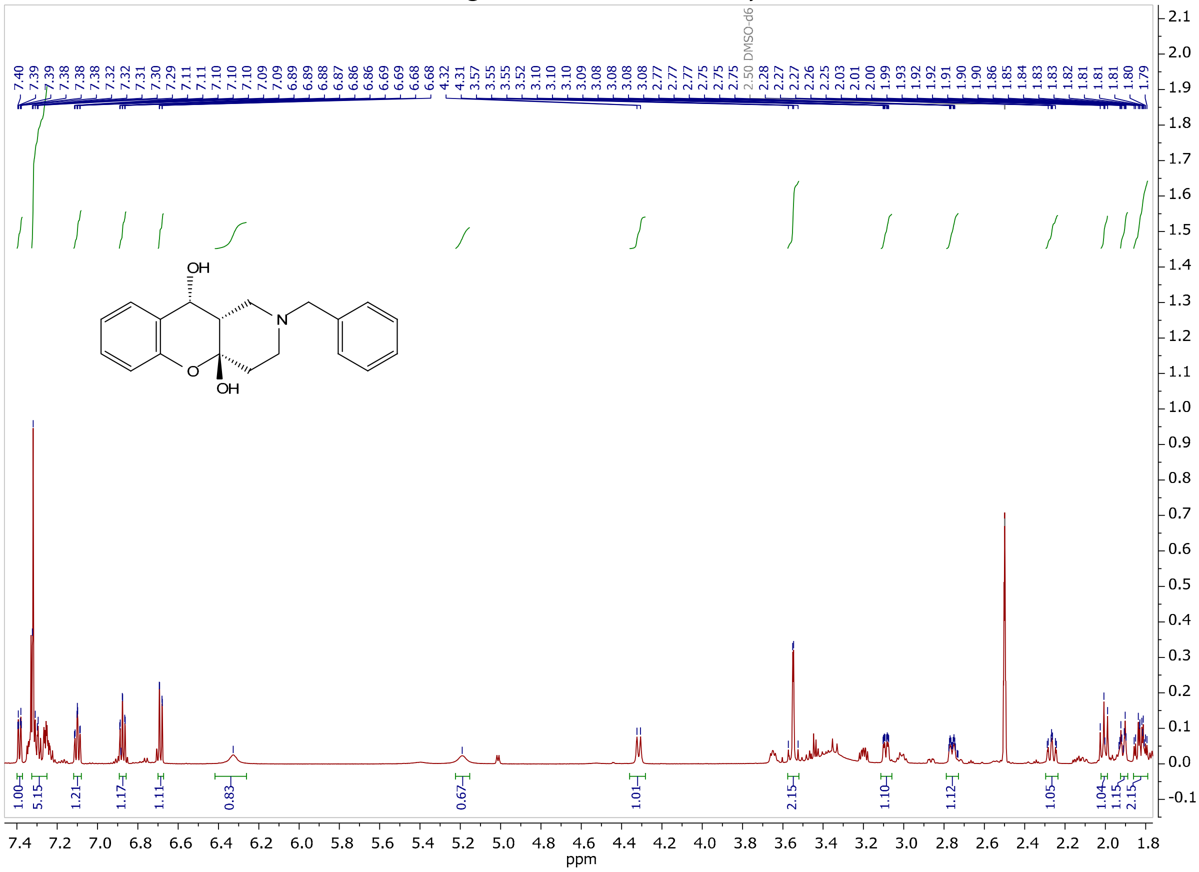

Figure S40. ^{13}C NMR data of **8e**

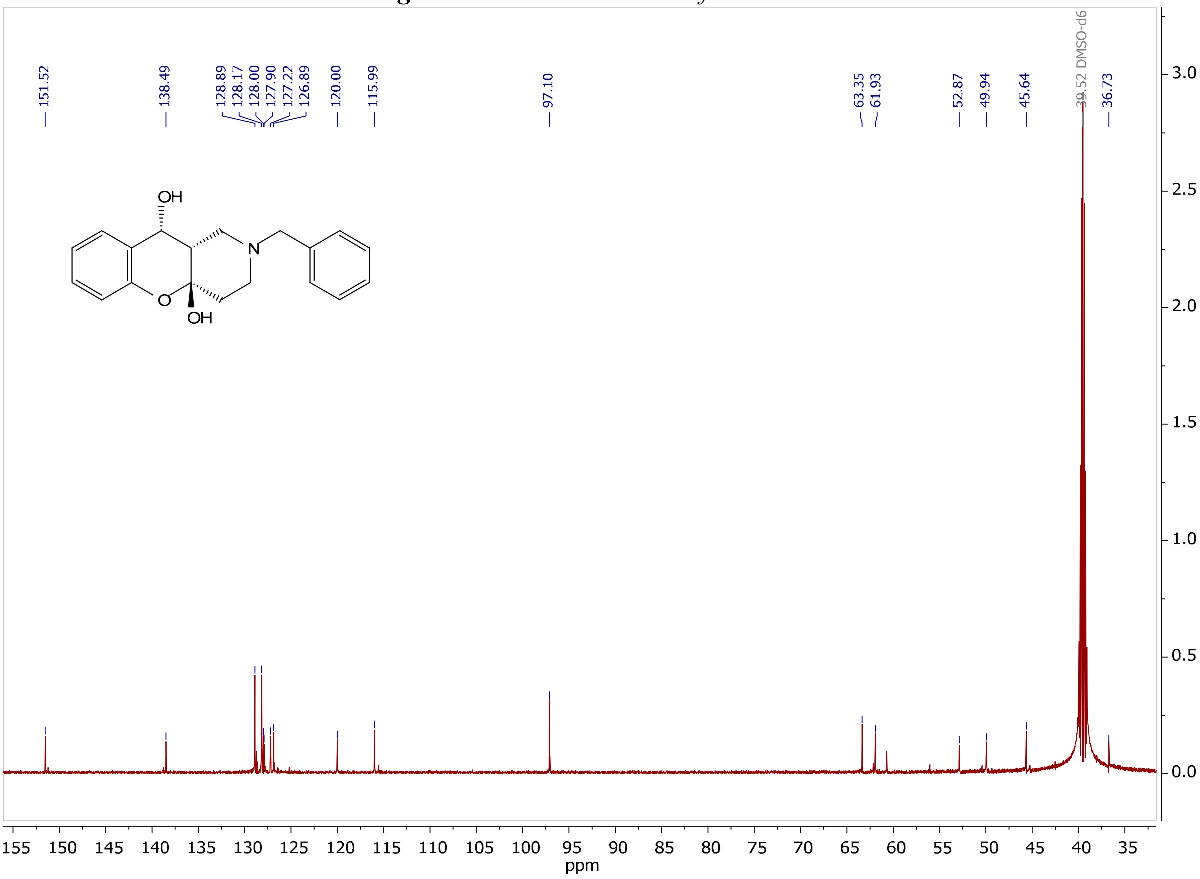

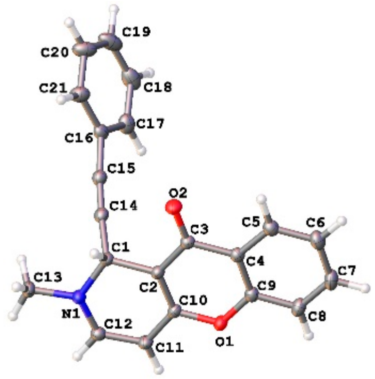

Crystal structure of compound **3a**.

Table S1. Crystal data and structure refinement for **3a**.

Empirical formula	$C_{21}H_{15}NO_2$
Formula weight	313.34
Temperature/K	120
Crystal system	monoclinic
Space group	$P2_1/c$
a/Å	11.5826(13)
b/Å	12.6646(15)
c/Å	11.4550(15)
α/°	90
β/°	111.547(4)
γ/°	90
Volume/Å^3	1562.9(3)
Z	4
ρ_{calc}g/cm^3	1.332
μ/mm^{-1}	0.086
F(000)	656.0
Crystal size/mm^3	0.3 × 0.25 × 0.22
Radiation	MoKα (λ = 0.71073)
2Θ range for data collection/°	3.78 to 60.952
Index ranges	$-11 \le h \le 16$, $-18 \le k \le 18$, $-16 \le l \le 13$
Reflections collected	12185
Independent reflections	4728 [R_{int} = 0.0190, R_{sigma} = 0.0234]
Data/restraints/parameters	4728/0/218
Goodness-of-fit on F^2	1.035
Final R indexes [I>=2σ (I)]	R_1 = 0.0458, wR_2 = 0.1217
Final R indexes [all data]	R_1 = 0.0567, wR_2 = 0.1318
Largest diff. peak/hole / e Å^{-3}	0.38/-0.21

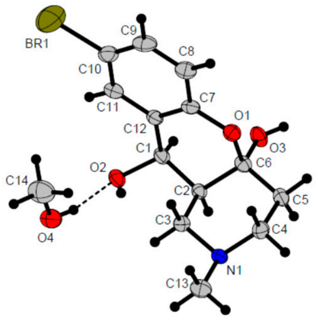

Crystal structure of compound **7c**.

Table S2. Crystal data and structure refinement for ***7c***.

Empirical formula	$C_{14}H_{20}BrNO_4$	
Formula weight	346.22	
Temperature	295(2) K	
Wavelength	1.54186 Å	
Crystal system	Monoclinic	
Space group	P 21/c	
Unit cell dimensions	a = 12.1496(3) Å	a= 90°.
	b = 8.7615(2) Å	b= 96.911(2)°.
	c = 14.4279(3) Å	g = 90°.
Volume	1524.67(6) $Å^3$	
Z	4	
Density (calculated)	1.508 Mg/m^3	
Absorption coefficient	3.794 mm^{-1}	
F(000)	712	
Theta range for data collection	3.665 to 68.386°.	
Index ranges	-14<=h<=14, -10<=k<=10, -11<=l<=17	
Reflections collected	15502	
Independent reflections	2676 [R(int) = 0.0719]	
Completeness to theta = 67.686°	96.2 %	
Refinement method	Full-matrix least-squares on F^2	
Data / restraints / parameters	2676 / 1 / 195	
Goodness-of-fit on F^2	0.837	
Final R indices [I>2sigma(I)]	R1 = 0.0340, wR2 = 0.0500	
R indices (all data)	R1 = 0.1065, wR2 = 0.0715	
Extinction coefficient	0.00057(5)	
Largest diff. peak and hole	0.209 and -0.242 e.$Å^{-3}$	

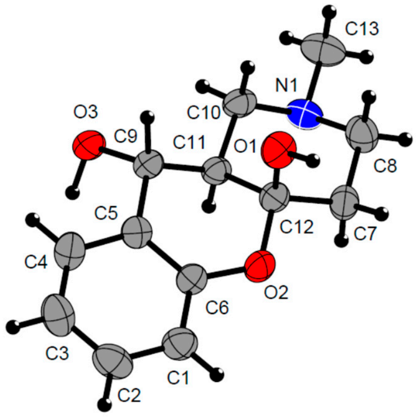

Crystal structure of compound **8a**.

Table S3. Crystal data and structure refinement for **8a**.

Empirical formula	$C_{13}H_{17}NO_3$	
Formula weight	235.28	
Temperature	295(2) K	
Wavelength	0.71073 Å	
Crystal system	orthorhombic	
Space group	P 21 21 21	
Unit cell dimensions	a = 9.8484(9) Å	a= 90°.
	b = 10.834(1) Å	b= 90.°.
	c = 10.7793(7) Å	g = 90°.
Volume	1160.78(18) Å^3	
Z	4	
Density (calculated)	1.346 Mg/m^3	
Absorption coefficient	0.096 mm^{-1}	
F(000)	504	
Theta range for data collection	3.76 to 30.68°.	
Index ranges	-12<=h<=14, -8<=k<=13, -7<=l<=14	
Reflections collected	3489	
Independent reflections	2462 [R(int) = 0.027]	
Refinement method	Full-matrix least-squares on F^2	
Data / restraints / parameters	2462 / 0/ 167	
Goodness-of-fit on F^2	1.017	
Final R indices [I>2sigma(I)]	R1 = 0.0568, wR2 = 0.0719	
Largest diff. peak and hole	0.200 and -0.279 e.Å^{-3}	
